# Chiral self-assembly of cellulose nanocrystals is driven by crystallite bundles

**DOI:** 10.1038/s41467-022-30226-6

**Published:** 2022-05-12

**Authors:** Thomas G. Parton, Richard M. Parker, Gea T. van de Kerkhof, Aurimas Narkevicius, Johannes S. Haataja, Bruno Frka-Petesic, Silvia Vignolini

**Affiliations:** Yusuf Hamied Department of Chemistry, University of Cambridge, Lensfield Road, Cambridge, CB2 1EW UK

**Keywords:** Self-assembly, Colloids, Liquid crystals, Photonic crystals, Bioinspired materials

## Abstract

The transfer of chirality across length-scales is an intriguing and universal natural phenomenon. However, connecting the properties of individual building blocks to the emergent features of their resulting large-scale structure remains a challenge. In this work, we investigate the origins of mesophase chirality in cellulose nanocrystal suspensions, whose self-assembly into chiral photonic films has attracted significant interest. By correlating the ensemble behaviour in suspensions and films with a quantitative morphological analysis of the individual nanoparticles, we reveal an inverse relationship between the cholesteric pitch and the abundance of laterally-bound composite particles. These ‘bundles’ thus act as colloidal chiral dopants, analogous to those used in molecular liquid crystals, providing the missing link in the hierarchical transfer of chirality from the molecular to the colloidal scale.

## Abstract (web summary)

Chirality transfer across length-scales is an intriguing phenomenon but connecting the properties of individual building blocks to the emergent features of their resulting large-scale structure remains challenging. Here, the authors investigate the origins of mesophase chirality in cellulose nanocrystal suspensions, whose self-assembly into chiral photonic films has attracted significant interest.

## Introduction

The chiral self-assembly of nanoscale building blocks is a universal phenomenon that demonstrates the emergence of large-scale structures from the properties of individual sub-units and offers a way to produce functional materials with bespoke properties^1,2^. In many self-organising colloidal systems such as amyloid fibrils and DNA origami filaments, the emergence of chirality in the mesophase can be directly correlated to the well-defined chiral morphology of individual assembling elements or their bending properties^3–6^. In general, however, directly tracking the transfer of chirality across length-scales remains a significant challenge, both for model colloids such as rod-like viruses and in systems of practical relevance^7^.

**Fig. 1:**
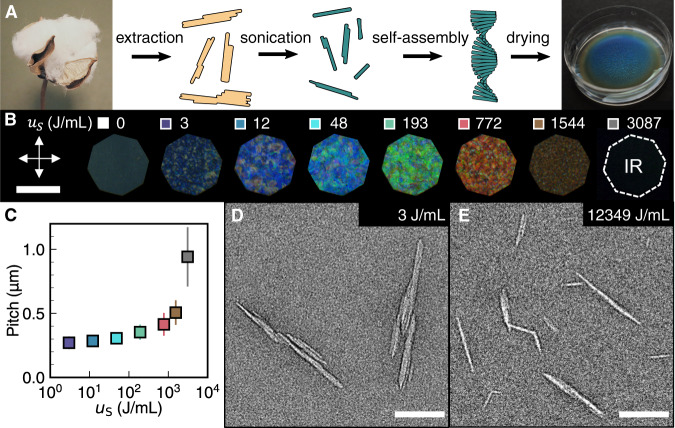
Tuning the colour of photonic CNC films with sonication. **A** Formation of structurally coloured films via chiral self-assembly of CNCs extracted from cotton. Petri dish size 35 mm. Cotton image source^49^: **B** Photonic CNC films of increasing sonication dose (${u}_{s}$), as viewed under crossed polarisers. Square panels next to each dose value indicate the colour code used for other plots. IR infrared. Scale bar 100 μm. **C** Pitch increase with sonication dose, as inferred from cross-sectional scanning electron microscopy. Error bars indicate standard deviation of 30 measurements. **D**, **E** Examples of TEM images of CNCs, showing their composite morphology. Scale bar 100 nm.

Cellulose nanocrystals (CNCs) are bio-sourced nanoparticles that spontaneously self-assemble in colloidal suspension to form a left-handed chiral nematic (cholesteric) phase (Fig. 1A)^8–10^. While their predisposition to self-assemble has been exploited as a chiral scaffold^11^ or to produce sustainable photonic colourants^12,13^, the origin of their mesophase chirality remains a highly debated topic. Individual cellulose Iβ crystallites are elongated and have a propensity to twist about their long axis^14–16^, a property ultimately arising from the molecular chirality of the β-1,4-D-glucose repeating unit^17–19^, but it is unclear how (or if) this subtle twist is sufficient to induce the chirality of the mesophase^20^. Moreover, the CNC population is highly polydisperse in particle size and shape^21^, which complicates attempts to attribute the chirality of the mesophase to specific features of individual particles.

By applying ultrasonication to controllably tune the size and shape of the CNCs and performing detailed nanoparticle shape metrology, here we establish quantitative relationships between the colloidal liquid crystalline behaviour and the morphological distribution of individual particles. This analysis reveals that a sub-population of composite particles, which we refer to as ‘bundles’, acts as colloidal chiral dopants, analogous to those used in molecular liquid crystals to transform a nematic phase into a cholesteric phase of a desired chiral strength^22^. As such, this ubiquitous but often ignored component of CNC suspensions is essential for the transfer of chirality across length-scales.

## Results

### Tuning CNC self-assembly with sonication

The strength of the chiral interactions in CNC suspensions can be accessed by measuring the chiral nematic pitch (defined as the period of one full rotation of the helicoidal structure), with weaker interactions corresponding to a larger pitch. While the pitch in suspension is typically on the order of 5–50 μm, compression of the structure upon evaporation of the solvent results in periodicity of 250–500 nm in dried films, giving rise to structural colour in the visible range^9^. The visual appearance of photonic CNC films is commonly tuned by treating the initial suspension with high-intensity ultrasound (hereafter referred to as ‘sonication’), which causes a red-shift in the colour of the films by increasing the chiral nematic pitch in suspension^23^. However, the mechanism by which sonication weakens the chiral interactions between individual CNCs is unclear.

To explore the effect of sonication on CNC self-assembly, we produced an initial CNC suspension by sulfuric acid hydrolysis of cotton (Methods) and applied sonication over a wide range of doses (*u*_*S*_ = 0–12 kJ/mL) to produce photonic films with colour across the visible range (Fig. 1B). Importantly, the sonication dose is expressed as caloric energy per suspension volume (J/mL), as we observed that consistent results could only be obtained using this unit (see Supplementary Fig. 1). The pitch, as measured by cross-sectional scanning electron microscopy (SEM), was found to increase with sonication dose (Fig. 1C, Supplementary Fig. 2). For photonic films with visible structural colour, the pitch values from cross-sectional SEM are consistent with the peak wavelengths from optical spectroscopy (Supplementary Fig. 3).

Identifying the origin of the pitch increase is challenging due to the numerous potential effects of sonication on individual particles, both physical (e.g. size, aspect ratio) and chemical (e.g. surface charge)^23^. By careful control of the suspension conditions after sonication (e.g. ionic strength) and characterisation of suspension properties (e.g. surface charge, pH), we concluded that the observed increase in pitch could only arise from sonication-induced changes in particle size and shape (see Supplementary Figs. 4–5 and associated discussion), suggesting that the chiral interaction between CNCs is fundamentally entropic. This conclusion is consistent with reports of chiral nematic phase formation in refractive index-matching apolar solvents, such as toluene, where electrostatic and van der Waals interactions are suppressed^24^. However, ensemble size properties such as hydrodynamic diameter (Supplementary Fig. 1) or particle cross-section (Supplementary Fig. 6) tend to constant values at high sonication dose, whereas the pitch diverges in this limit, suggesting that the crucial morphological changes are more subtle and require more detailed characterisation of individual particles.

The morphology of individual particles can be observed using transmission electron microscopy (TEM), as exemplified in Fig. 1D and Fig. 1E, where the preparation of TEM grids was optimised to remove the physical overlap of distinct particles (see Methods). We therefore define a CNC particle, in the context of this study, as any continuous nano-object observed in TEM images (see Supplementary Methods for further discussion). While all CNC suspensions exhibit considerable variation in particle size and shape, there are clear morphological trends with sonication. At a low dose, the CNC population contains a large number of irregular-shaped particles that are composed of multiple elongated sub-units bound perpendicular to their long axis (Fig. 1D). The dimensions of the sub-units are consistent with those expected for individual cellulose crystallites, which indicates that the CNC population is a mixture of isolated elementary crystallites and multi-crystallite composite particles, in agreement with previous reports for CNCs derived from wood pulp and cotton^25,26^. At higher sonication doses, the fragmentation of composite particles leads to a higher proportion of isolated cellulose crystallites (Fig. 1E).

The sonicated-induced release of individual cellulose crystallites is noteworthy, as their right-handed axial twist is often cited as the likely origin of mesophase chirality in CNC suspensions^16,27^, and is supported by simulations of monodisperse hard polyhedral particles with a fixed axial twist^28^. However, if the mesophase chirality arose directly from the twisted morphology of individual cellulose crystallites, the pitch would be expected to remain unchanged or even decrease as the crystallites are liberated from achiral composite particles by sonication. In practice the opposite trend is observed, suggesting that the crystallite twist is not, by itself, sufficient to explain the mesophase chirality. This conclusion led us to consider the possible role of composite particles in CNC self-assembly.

### Tracking the morphological evolution of CNCs

**Fig. 2:**
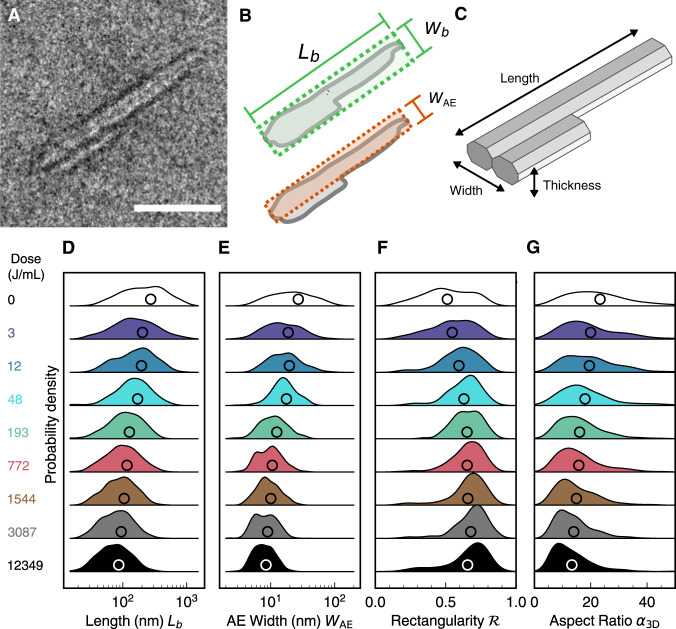
Revealing the 3D morphological changes due to sonication by nanoparticle shape metrology. **A** Example of a CNC particle in a TEM image. Scale bar 50 nm. **B** Definition of the box length, box width and area-equivalent (AE) width. **C** Schematic of the 3D morphology of the particle in (**A**), with the length, width and thickness dimensions indicated. **D**–**G** Distributions in particle size and shape properties for box length (**D**), AE width (**E**), rectangularity (**F**) and 3D aspect ratio (**G**), plotted in vertical stacks to facilitate comparison of statistical features between samples. Open circles indicate mean values.

Historically, composite CNC particles have often been overlooked when manually extracting morphological properties from TEM images, on the assumption that they are an artefact of sample preparation. Having first verified that multi-crystallite particles are a native component of the particle population using cryogenic TEM images of dilute CNC suspensions (Supplementary Fig. 7), we sought morphological properties to characterise particle size and shape. Individual cellulose crystallites have a regular, highly elongated morphology, making it appropriate to represent their outlines as simple model shapes such as rectangles or ellipses. However, to accurately capture the complex, irregular shapes of many CNCs (Fig. 2A), such approximations are insufficient. For instance, fitting the particle shape to a bounding rectangle of dimensions *L*_*b*_ and *W*_*b*_ over-estimates the projected particle area (*A* ≤ *L*_*b*_*W*_*b*_) and under-estimates the aspect ratio (Fig. 2B, C), making it difficult to correlate subtle changes in particle morphology with ensemble behaviour.

We therefore developed an in-depth morphological analysis protocol and applied it to CNC outlines obtained from TEM images (see Methods and Supplementary Methods), to quantify the evolution in particle size and shape with sonication dose. This detailed nanoparticle metrology allows us to measure more appropriate size properties, such as the area-equivalent (AE) width, *W*_*AE*_ = *A/L*_*b*_, which better represents the average width of elongated but irregular particles like those found in CNC suspensions.

The sonication-induced fragmentation of CNCs can be understood by examining the change in the key morphological properties. This is exemplified by Fig. 2D, E, which shows the size distributions of *L*_*b*_ and *W*_*AE*_ for CNC suspensions exposed to a range of sonication doses. The general trend is a decrease in particle dimensions with sonication dose, which we also observed for other common size properties such as area or perimeter (Supplementary Fig. 8). Notably, while the mean box length (Fig. 2D) decreases gradually with sonication dose, the mean AE width (Fig. 2E) converges towards a limiting value of 8 nm, comparable to that expected for a single cellulose crystallite^25^. Furthermore, the narrowing of the length and width distributions in Fig. 2D, E with sonication dose indicates a decrease in the coefficient of variation (i.e., standard deviation over mean, a measure of relative polydispersity).

Alongside size properties, this analysis also allows us to quantify size-invariant shape properties such as particle rectangularity, defined as

1 $${{{{{\mathscr{R}}}}}}=A/{L}_{{{\mbox{b}}}}{W}_{{{\mbox{b}}}}={W}_{{{\mbox{AE}}}}{/W}_{{{\mbox{b}}}}.$$

The rectangularity ranges from 0 to 1, with ${{{{{\mathscr{R}}}}}}=1$ for a particle with an ideal rectangular profile. The increase in mean particle rectangularity (Fig. 2F) provides robust statistical evidence of the simplification of particle shape with sonication dose, which is consistent with the qualitative observation of fragmentation leading to individual parallelepipedic crystallites. Further evidence of this trend is readily obtained from other shape properties, such as particle convexity and solidity (Supplementary Fig. 9).

Another fundamental shape property for elongated particles is the aspect ratio (i.e. the ratio of longer and shorter particle dimensions). However, accurately measuring the aspect ratio of CNCs is non-trivial due to their irregular shapes and their non-circular cross-sections, which are not visible in TEM images. For individual cellulose crystallites, the cross-section perpendicular to the long axis is known to be approximately rectangular (Fig. 2C), with a width and thickness of around 6–8 nm for crystallites derived from cotton^25,29^. More generally, the 3D morphology of CNC particles can be probed by atomic force microscopy (AFM) or small-angle scattering, with previous studies indicating that many CNCs are composed of laterally bound crystallites into “bundled” or “raft-like” structures^25,30,31^. By combining TEM and AFM analysis, we estimated the mean thickness 〈*T*〉 of each particle observed in TEM (see Supplementary Figs 10–14 and associated discussion), which allows us to predict the effective 3D aspect ratio ${\alpha }_{3D}={L}_{b}/\sqrt{{W}_{{{\mbox{AE}}}}\left\langle T\right\rangle }$, as opposed to the apparent 2D aspect ratio obtained from TEM (i.e. ${\alpha }_{2D}={L}_{b}/{W}_{b}$). The 3D aspect ratio decreased substantially with sonication dose (Fig. 2G), while 2D aspect ratio values showed no clear trend (Supplementary Fig. 9). The relevance and accuracy of ${\alpha }_{3D}$ is demonstrated by its ability to accurately estimate ensemble size properties such as the average hydrodynamic diameter and particle cross-section from the morphology of individual particles (Supplementary Fig. 15). The 3D morphological analysis using to estimate ${\alpha }_{3D}$ also gave access to a variety of other important shape properties, such as the aspect ratio of the particle cross-section (${\alpha }_{{{\mbox{XS,AE}}}}={W}_{{{\mbox{AE}}}}/\left\langle T\right\rangle$) and the 3D isoperimetric quotient (Supplementary Fig. 10).

### Quantifying the effect of morphology on CNC mesophases

The decrease in ${\alpha }_{3D}$ with sonication is notable because of its predicted effect on the lyotropic phase behaviour of CNC suspensions. In the limit of infinite dilution, CNC suspensions are isotropic (I), with no correlation in particle orientations. Above a critical CNC volume fraction *ϕ*_0_, the chiral nematic (N*) phase is first observed in coexistence with the isotropic phase. The relative proportion of the N* phase grows with concentration until the suspension becomes fully chiral nematic at a second critical volume fraction *ϕ*_1_. Notably, the critical concentrations (*ϕ*_0_*,ϕ*_1_) are predicted to be inversely proportional to the particle aspect ratio^32^. At higher concentrations, equilibration can be inhibited by the onset of kinetic arrest (KA) with the mechanism of KA (whether glass transition or gelation) dependent on the colloidal conditions^9,33^.

**Fig. 3:**
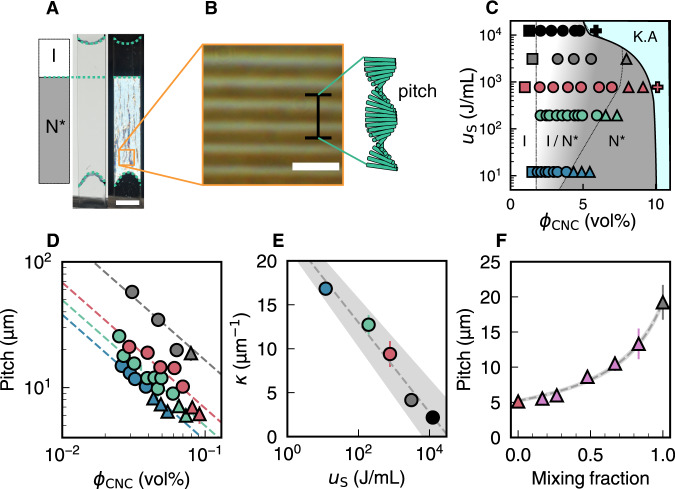
Quantitative analysis of chiral self-assembly behaviour. **A** Macroscopic phase separation of a biphasic CNC suspension in a sealed rectangular glass capillary. Vertical separation into isotropic (I) and chiral nematic (N*) phases is evident when viewed under crossed polarisers (right photograph). Scale bar 5 mm. **B** Polarised optical microscopy of the N* phase from (**A**). The periodicity of the fingerprint pattern corresponds to a 180° rotation of the chiral nematic order and is equal to half the pitch. Scale bar 10 μm. **C** Phase diagram versus CNC concentration and sonication dose, with the isotropic (I, squares), biphasic (I/N*, circles), chiral nematic (N*, triangles) and kinetically arrested (K.A., crosses) phases indicated. **D** Variation in pitch with CNC concentration for biphasic and chiral nematic suspensions (symbols match the phase diagram (**C**)). Dotted lines indicate linear fitting. **E** Decrease in chiral strength κ with sonication dose. Error bars indicate standard deviation of 30 measurements. Grey line indicates empirical logarithmic relation. Grey shaded region indicates uncertainty in fitting. **F** Pitch of mixtures of ‘low dose’ (772 J/mL, red) and ‘high dose’ (3087 J/mL, brown) chiral nematic suspensions at equal CNC volume fraction. Error bars indicate standard deviation of 30 measurements. Grey line indicates fitting to chiral dopant model. Grey shaded region indicates uncertainty in fitting.

To investigate the correlations between CNC phase behaviour and particle morphology, we prepared sealed capillaries containing suspensions at a range of concentrations and sonication doses and allowed them to reach equilibrium (Fig. 3A). As shown in Fig. 3C, we found that the onset of the biphasic concentration range (*ϕ*_0_) was not strongly affected by sonication, while the onset of the fully chiral nematic phase (*ϕ*_1_) was delayed. These trends are broadly consistent with the decrease in mean 3D aspect ratio with sonication dose (Supplementary Fig. 16). At the highest dose (*u*_*S*_ = 12 kJ/mL), sonication advanced the onset of kinetic arrest, with the low ionic strength of the suspensions and microscale appearance being consistent with a repulsive colloidal glass^33^. This observation may be related to the greater effective volume fraction in suspension for these highly fragmented particles^34^.

Importantly, the capillaries also enable the measurement of the chiral nematic pitch in suspension, which appears as a periodic “fingerprint pattern” visible by polarised optical microscopy (Fig. 3B). At concentrations below the onset of the kinetic arrest, the equilibrium pitch was found to be inversely proportional to the CNC volume fraction at all sonication doses (Fig. 3D). For samples at comparable CNC concentrations, we found that sonication universally led to a larger pitch, indicating that the red-shift observed in dried films originates from a larger pitch in the suspension, and not from variation in the compression of the chiral nematic structure upon drying. These observations can be formulated as a linear relationship between the chiral nematic wavevector, *q* = 2*π/P*, and the CNC volume fraction, *ϕ*:

2 $$q=\kappa \phi ,$$

where *κ*(*u*_*S*_) is a dose-dependent property that captures the strength of the chiral interactions in the suspension. Expressing the mesophase chirality in terms of this chiral strength *κ*, instead of the pitch, enables direct comparison between values at a wide range of concentrations. We observe that the chiral strength decreases with sonication dose according to an empirical logarithmic fit (Fig. 3E).

Remarkably, the linear scaling in Eq. (2) is reminiscent of the behaviour of chiral doping in molecular liquid crystals^22^. For a nematic liquid crystal doped by chiral molecules at a volume fraction *ϕ*_*d*_, the wavevector of the resulting chiral nematic phase is

3 $$q=4\pi {\beta }_{V}{\phi }_{d},$$

where *β*_*V*_ is the helical twisting power (HTP) of the chiral dopants. The scaling behaviour in CNC suspensions thus suggests that only a small proportion of the CNC population actively contributes to the mesophase chirality. We hypothesised that a specific sub-population of the composite CNC particles act as chiral dopants, while isolated elementary crystallites are functionally achiral and tend to form a nematic (i.e. non-cholesteric) phase. The decrease in chiral strength with sonication dose can then be attributed to the fragmentation of these composite particles into elementary crystallites.

To validate this hypothesis, we mixed two CNC suspensions of different sonication doses at constant CNC concentration. We chose ‘low-dose’ (772 J/mL) and ‘high-dose’ (3087 J/mL) samples with substantially different pitch values at the chosen concentration (*ϕ* = 7.9 vol%) but comparable mean particle length and AE width. This concentration was chosen to ensure both samples were fully chiral nematic, ruling out any possible complications due to fractionation within a biphasic suspension^34,35^. The equilibrium pitch values for mixtures of low and high-dose samples (Fig. 3F) are consistent with the chiral dopant hypothesis, which predicts a harmonic pitch average for the mixtures (i.e., a linear mixing in terms of the corresponding wavevectors *q*), due to variation of the abundance of dopants according to Eq. 3. Notably, the trend in Fig. 3F is not consistent with pitch variation arising from bulk suspension properties such as ionic strength, where the resulting pitch would be a linear interpolation of the pitches of the two original samples, as previously proposed^23^.

### Relating CNC mesophase chirality to the abundance of bundles

The evolution of the pitch in CNC mixtures reported in Fig. 3F motivated us to re-examine the CNC morphological analysis for evidence of a sub-population acting as chiral dopants. The morphological analysis presents a complex picture of the evolution of the particle population with increasing sonication dose. For instance, in Fig. 2F a low-rectangularity (${{{{{\mathscr{R}}}}}}$) tail was observed at a low dose, which disappeared at intermediate doses but re-emerged at high doses. Direct inspection of TEM images revealed that low-dose suspensions (*u*_*S*_ ≤ 48 J/mL) had a considerable number of large, irregularly shaped particles with low rectangularity (i.e. shapes that were poorly approximated by a bounding rectangle). The proportion of these irregular particles decreased rapidly as the sonication dose increased. In contrast, much higher doses (*u*_*s*_ ≥ 772 J/mL) led to the occurrence of particles that appeared to be elementary crystallites distorted by sharp kinks. Although these kinked particles also showed low rectangularity, they were markedly thinner than the low-rectangularity particles observed at lower doses. Furthermore, their morphology was similar to previous reports for CNCs extracted from tunicate or for CNCs produced by sonicating cellulose nanofibres^25,36^, leading us to conclude that the kinked particles were elementary crystallites that had been mechanically damaged by extensive sonication.

A plausible model of chiral composite particles is a ‘twisted raft’ or ‘propeller’ configuration, where crystallites are bound together perpendicular to their long axes, with a twist about the binding axis arising from the axial twist on the crystallite sub-units. A recent simulation study has demonstrated that an ensemble of hard homochiral ‘twisted raft’ particles will form a chiral nematic phase, with pitch values comparable to CNCs suspended in apolar solvents^37^. More generally, among the diversity of particle shapes observed in CNC samples, we anticipate an enantiomeric excess of composite particles with a right-handed twisted morphology with the symmetry-breaking arising from the weak right-handed twist on individual crystallites. Previous studies applying cryogenic electron tomography to individual CNCs have observed chiral composite particles, but the practical limitations of this technique have made it difficult to establish a net chiral bias^38^. It is difficult to discern the 3D chiral morphology of CNCs from the individual 2D projections provided by TEM images, and the drying of the sample onto the TEM grid leads to distortion of the chiral structure^16^. However, the kind of structures necessary for chiral doping should be distinguishable from disordered aggregates and individual cellulose crystallite (whether native or kinked) by their morphological properties. In this regard, drying the sample onto a TEM (or AFM) grid has the advantage of flattening particles onto the grid, allowing their dimensions to be measured without relying on estimations from the projected 2D shape^25^.

We therefore classified CNCs into different sub-populations according to their AE width and rectangularity, with threshold boundaries defined at *W*_*AE*_ = 17 nm and ${{{{{\mathscr{R}}}}}}$ = 0.40. The width threshold was chosen so that particles above this threshold are unquestionably composite particles (i.e. *W*_*AE*_ > 2 *W*_*C*_, where *W*_*C*_ is the average width of a single crystallite), while the rectangularity threshold was chosen based on the shape of the distributions in Fig. 2F. This filtering by two morphological properties leads to four particle classes. For convenience, each class is alliteratively named according to the morphology of a typical particle: (A) Aggregates (*W*_*AE*_ high, ${{{{{\mathscr{R}}}}}}$ low), (B) Bundles (*W*_*AE*_ high, ${{{{{\mathscr{R}}}}}}$ high), (C) Crystallites (*W*_*AE*_ low, ${{{{{\mathscr{R}}}}}}$ high) and (D) Distorted (kinked) crystallites (*W*_*AE*_ low, ${{{{{\mathscr{R}}}}}}$ low). From qualitative inspection of the TEM images, we note that the Aggregate and Bundle sub-populations contain wide, composite CNC particles formed of multiple elementary cellulose crystallites laterally connected together (i.e. connected along the plane perpendicular to their long axes). The Aggregates are distinguished from Bundles by their disordered morphology, as expressed by the particle rectangularity. Among the thinner CNC particles, the Crystallite sub-population is distinguished from a fourth sub-population of “Distorted crystallites”, as the latter have a kinked shape and as such appear to be mechanically damaged. The labelling of the classes does not perfectly describe every particle in each sub-population (especially for the Crystallite sub-population), but captures the essential features. The wide array of available morphological properties offers many alternative ways to divide CNCs into multiple classes (e.g. based on the aspect ratio of the particle cross-section, Supplementary Fig. 10). The classification above was chosen as a simple but effective example that relies purely on properties directly observed from TEM images, without relying on the estimation of the particle thickness or other 3D properties not accessible by TEM.

**Fig. 4:**
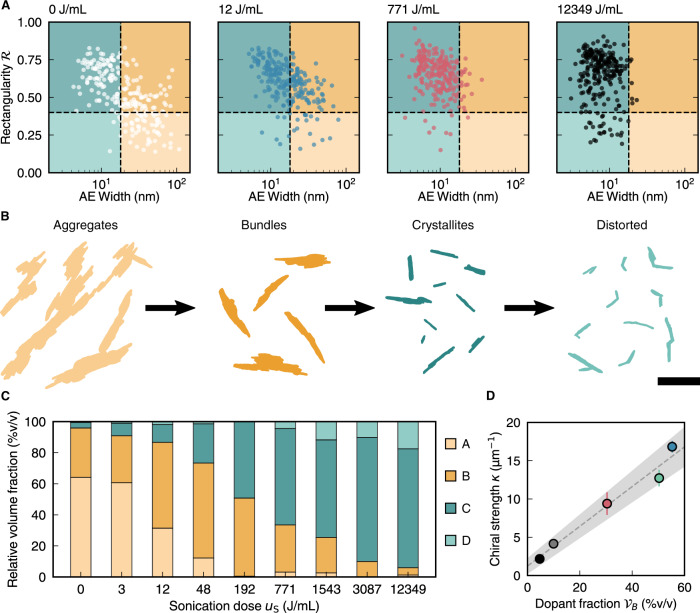
Relating the CNC mesophase chirality to the abundance of crystallite bundles. **A** Classification of CNC particles into four sub-populations based on AE width and rectangularity. Scatter plots of AE width versus rectangularity are shown for four sonication doses, illustrating the evolution of the system under sonication. **B** Representative examples of each particle sub-population. Evolution under sonication indicated by black arrows. Scale bar 200 nm. **C** Relative volume fraction of each particle sub-population versus sonication dose. **D** Modulation of chiral strength with relative dopant volume fraction. Error bars indicate standard deviation of 30 measurements. Grey dotted line indicates linear correlation. Grey shaded region indicates uncertainty in fitting.

Figure 4A displays the population distributions of CNC particles at selected sonication doses. The overall trend with sonication is a migration of the particle distribution anticlockwise in the ${W}_{{AE}}{{{{{\mathscr{-}}}}}}{{{{{\mathscr{R}}}}}}$ parameter space, from Aggregates (bottom right) through Bundles (top right) and Crystallites (top left) to Distorted crystallites (bottom left). This evolution in particle morphology is represented in Fig. 4B, which shows typical particles in each sub-population. We then quantified the relative volume fraction (${{{{{{\mathscr{V}}}}}}}_{A}$, ${{{{{{\mathscr{V}}}}}}}_{B}$, etc.) of each particle sub-population (see Supplementary Information section 11), which allowed us to track the evolution of the CNC population with sonication, as shown in Fig. 4C. For completeness, the evolution of the relative number fractions of each sub-population can be found in Supplementary Fig. 17, and histograms for various morphological properties of the sub-populations can be found in Supplementary Figs. 18–20.

The trends in Fig. 4C match the qualitative observations of Fig. 4A, and are consistent with the ensemble size measurements such as suspension turbidity or hydrodynamic diameter (Supplementary Fig. 15). While ensemble measurements are only an indirect measure of particle dimensions, any morphological differences observed between samples are highly robust. In this case, a marked decrease in turbidity (measured by UV-vis spectroscopy) and hydrodynamic diameter (measured by dynamic light scattering) was observed, with both displaying a rapid initial decrease at low sonication doses and then a more gradual decrease at higher doses (Supplementary Fig. 15). In terms of our particle classification, these observations confirm that the Aggregate and Bundle sub-populations are initially dominant and that, upon sonication, Aggregates are readily converted into smaller structures while the Bundles are more persistent and require higher doses to be broken down into individual elementary crystallites.

Having validated the evolution of particle morphology by comparing ensemble measurements, we considered whether a specific sub-population act as chiral dopants. The dopant concentration can be related to the total CNC concentration by defining a relative dopant volume fraction *χ*, which can be obtained by combining Eqs. (2) and (3):

4 $$\chi =\,{\phi }_{d}/\phi .$$

By comparing sub-population trends in Fig. 4C to the corresponding chiral strength values in Fig. 3E, we identified the Bundle sub-population as potential chiral dopants (i.e. $\chi ={{{{{{\mathscr{V}}}}}}}_{B}$), as expected by the ‘twisted raft’ model. We discounted the Aggregates as potential chiral dopants because this sub-population is absent at moderate to high sonication doses, and their morphology is highly irregular. Chirality transfer is expected to be stronger between particles with compatible morphological properties (e.g., isoperimetric quotient), and in this respect the Crystallites are much closer to the Bundles than to the Aggregates (see histograms in Supplementary Information Fig. 20)^39^. Furthermore, chirality transfer requires a non-circular particle cross-section, and the Bundles are found to have a substantially larger cross-section aspect ratio than the Crystallite and Distorted crystallite sub-populations (Supplementary Fig. 19).

The chiral strength *κ*, extrapolated from the measurements of the volume fraction and the pitch, shows a clear positive trend with dopant abundance, as shown in Fig. 4D. It also increases linearly with *χ* at low Bundle abundance, in accordance with Eq. 4, implying a constant HTP at a high sonication dose. Notably, the chiral nematic wavevector *q* tends to zero at high sonication, supporting the conclusion that the crystallites are functionally achiral.

The limiting behaviour of Fig. 4D gives an estimate of the HTP per bundle volume fraction as *β*_*V*_ = –4 μm^−1^, where the negative sign is used to indicate left-handed helicoidal ordering. This value is comparable to those obtained for cholesteric colloidal systems of DNA origami filaments and amyloid fibrils, where the estimated helical twisting powers were ${|\beta }_{V}|$ ≈ 2 and ${|\beta }_{V}|$ ≈ 20 μm^−1^ respectively^3,4^. While the chiral dopant model is expected to be universally applicable to the mesophase behaviour of any CNC suspension, we expect the fundamental properties of the Bundle sub-population (i.e. helical twisting power and relative abundance) to vary slightly with cellulose source and extraction method. Interestingly, by considering the HTP per mass fraction (*β*_*w*_ *≈* –2.5 μm^−1^), a comparison can be made with previous reports of functionalised CNCs doping a molecular nematic mesophase (*β*_*w*_ *≈* –50 μm^−1^). The more effective inducement of chirality in the latter case can be attributed to the much smaller mesogen size and continuous short-range contact between the mesogens and chiral dopants^40^.

## Discussion

These results prompt a re-examination of the existing literature on the chiral self-assembly of CNCs. First, it has been shown that fractionation of CNC suspensions by phase separation leads to an accumulation of longer particles in the anisotropic phase, with a positive correlation between mean particle length and helical twisting power^12,34,35^. Here, we observed that the Bundle sub-population has a greater mean particle length and 3D aspect ratio than the Crystallites (Supplementary Fig. 18), and that the helical twisting power is proportional to the volume fraction of this sub-population relative to the total population. We therefore interpret the observed fractionation as an indication that Bundles preferentially enter the anisotropic phase. Second, our model is consistent with the observed decrease in pitch when increasing the ionic strength of CNC suspensions^41^, as greater screening of the electrostatic repulsion between particles allows them to come into closer proximity, where the entropic (i.e. short-range) chiral interaction can take effect. Finally, composite CNCs acting as chiral dopants offer a convincing and self-consistent explanation for the pitch increase upon sonication, in contrast to the previously proposed model of Beck et al.^23^, where the pitch increase was attributed to the release of tightly bound ions and an increase in the effective thickness of the CNC surface layer (Supplementary Information, section 4). Importantly, the increase in chiral nematic pitch due to sonication has been observed for suspensions of polysaccharide nanocrystals from numerous sources, including CNCs from cotton^23^ and wood pulp^13^, as well as chitin nanocrystals^42^, suggesting that the chiral dopant model can be applied to understanding the chirality of these systems^43^.

Despite the strong evidence of the chiral dopant model in this work, a detailed quantitative analysis of 3D CNC morphology sufficient to demonstrate an enantiomeric excess of chiral particles is still lacking. Although it has proven difficult to quantitatively assess the axial twist of individual crystallites using cryo-electron tomography^38^, this technique may be able to directly observe the chirality of multi-crystallite bundles, whose twisted 3D morphology should be more pronounced. At the same time, building upon recent work^37^, further simulation studies are warranted to investigate the influence of key morphological parameters, such as the number of crystallites per bundle. It is important to note that the chiral dopant model is widely applicable, regardless of the specific dopant shape, and is not limited to the specific composite particle shape (the ‘twisted raft’) assumed in this work.

The role of CNC bundles as chiral enhancers suggests a new paradigm for colloidal self-assembly, analogous to chiral dopants in molecular liquid crystals. It is difficult to manufacture microscale particles with a consistent chiral morphology, often requiring intensive top-down methods or expensive starting materials. Our results suggest that large-scale complex self-assembly could instead be achieved by adding a small fraction of precisely designed chiral seed particles into an achiral bulk phase produced by simpler methods.

The detailed nanoparticle metrology used in this work opens up a new avenue of enquiry for research on anisotropic bio-sourced nanomaterials. Beyond the role of chirality, nanocellulose formulations prepared from different sources and extraction methods exhibit considerable variation in performance^44,45^, which could be systematically analysed and correlated with the prevalence of specific morphological features. More generally, standardising the characterisation of nanoparticle morphology is a major outstanding challenge for nanomaterials research^46,47^. The particle analysis developed for this study, which is widely transposable to other systems, may help address this challenge.

## Methods

### Preparation of CNC suspensions

Cellulose nanocrystals were obtained by acid hydrolysis of cotton (60 g, Whatman No. 1 cellulose filter paper) in sulfuric acid (840 mL, 64 wt%, ≥95% analytical reagent grade, Fisher Scientific) at 64 °C under high mechanical stirring. The reaction was quenched after 30 min by dilution of the acid with deionised ice and water, and by immersion of the reaction vessel in an ice bath. Soluble cellulose residues and excess acid were removed by three rounds of centrifugation at 20,000 × *g* (30, 20 and 20 min, respectively) with the pellet redispersed in deionised water after each round. Excess ions were removed by dialysis against deionised water using MWCO 12–14 kDa membranes. This purification resulted in a 2.43 wt% CNC suspension, which was used as the starting batch for further treatment.

### Determination of CNC mass and volume fraction

The concentration of CNC suspensions was measured by a thermogravimetric method. Vials containing CNC suspensions were weighed before and after drying (>24 h at 60 °C) to determine the CNC mass fraction *μ*_*CNC*_. The CNC volume fraction *ϕ* was then estimated using the relation

$$\phi =\frac{{\mu }_{{CNC}}/{\rho }_{{CNC}}}{{\mu }_{{CNC}}/{\rho }_{{CNC}}+{\mu }_{w}/{\rho }_{w}}$$

where ${\mu }_{w}=1-{\mu }_{{CNC}}$ is the mass fraction of water and ${\rho }_{{CNC}}$ and ${\rho }_{w}$ are the mass densities of CNC and water (taken to be 1600 and 1000 kg m^−3^, respectively).

### Ultrasonication

The nanocrystal size was controlled by high-intensity sonication (Fisherbrand Ultrasonic disintegrator, 20 kHz, tip diameter 12.7 mm operating with a 2 s: 1 s ON:OFF cycle). Unless otherwise stated, samples were sonicated at 2 wt% CNC with a sample volume of 20 mL in a truncated centrifuge tube (Corning Falcon 50 mL), with the sonicator tip immersed to a depth of one-third of the sample volume. Samples were immersed in an ice bath throughout sonication to prevent sample heating, as desulfation of the CNCs surface charges has been observed in acidic suspensions at high temperatures^48^. For longer sonication treatments, the ice bath was replenished every five minutes.

### Transmission electron microscopy (TEM)

TEM samples were prepared by casting a droplet of 0.001 wt% CNC suspension in pH 3 sulfuric acid solution onto carbon-coated copper grid prepared by glow discharge and staining with uranyl acetate solution. TEM images were captured using a Talos F200X G2 microscope (FEI) operating at 200 kV and a CCD camera. The CNC outlines were traced in Fiji (ImageJ) software and analysed using the Shape Filter plug-in. As CNCs exhibit considerable size polydispersity, at least 250 particles were measured for each sample to ensure reliable statistics. Discussion of the tracing protocol is given in the Supplementary Methods.

### Preparation of photonic CNC films

To standardise the CNC suspensions after dialysis and ensure the optimal visual appearance of the final films, each suspension was diluted to 1.6 wt% CNC in 1.92 mm NaCl solution. Films were cast from 2.5 mL of each suspension into a 35 mm diameter polystyrene Petri dish and left to dry under ambient conditions (20 °C, 40–50% relative humidity), which typically took 36 h.

### Polarised optical microscopy

Optical microscopy was performed on a Zeiss Axio microscope, with a halogen lamp (Zeiss HAL100) as a light source using Koehler illumination. Images were captured in bright field reflection mode using a 10× objective (Nikon T Plan SLWD, NA 0.2) and recorded using CMOS camera (UI-3580LE-C-HQ, IDS). The white balance of the images was calibrated using a white Lambertian diffuser.

### Chiral nematic phase and pitch measurement

CNC suspensions at a range of concentrations were prepared by first concentrating the stock using a rotary evaporator (40 °C, pressure <40 mbar), then diluting to the desired final concentration using deionised water. Chiral nematic phase separation was observed in CNC suspensions at a range of concentrations. Glass capillaries were filled with the suspensions and sealed with UV epoxy (Norland Optical Adhesive 81), then left to equilibrate for at least 2 weeks. The anisotropic phase fraction was determined from the proportion of the suspension that appeared bright when viewed between crossed polarisers. The chiral nematic pitch of the anisotropic phase was measured as twice the periodicity of the fingerprint pattern observed in optical microscope images, which were collected using transmission illumination with crossed polarisers on a Zeiss Axioscope microscope equipped with a ×20 objective (Nikon T Plan SLWD, NA 0.3).

## Supplementary information

Supplementary Information

## Data Availability

All raw and processed data relating to this publication are freely available from the University of Cambridge data repository (10.17863/CAM.83319).
