# Supplementary material for: Chiral self-assembly of cellulose nanocrystals is driven by crystallite bundles
Source: Nat Commun. 2022 May 12;13:2657. doi: 10.1038/s41467-022-30226-6 (PMC9098854; doi:10.1038/s41467-022-30226-6)
Supplement: Supplementary file 1 — Supplementary Information [file 41467_2022_30226_MOESM1_ESM.pdf]

Supplementary Information for Chiral Self-Assembly of Cellulose Nanocrystals is Driven by Crystallite Bundles

Thomas G. Parton, Richard M. Parker, Gea T. van de Kerkhof, Aurimas Narkevicius, Johannes S. Haataja, Bruno Frka-Petesic, Silvia Vignolini*
Yusuf Hamied Department of Chemistry, University of Cambridge

*Corresponding author sv319@cam.ac.uk

Contents

1 Calibration of sonication dose by calorimetry **3**

2 Pitch measurement by SEM on CNC film cross-sections **4**

3 Pitch measurement by optical spectroscopy on CNC photonic films **5**

4 Discussion of other explanations of pitch increase induced by sonication **6**
4.1 Release of trapped ions 6
4.2 Surface charge and colloidal stability 7

5 Estimation of particle cross-section using turbidity spectra **9**

6 Evidence of bundles from cryoTEM imaging **12**

7 Definitions and distributions of morphological properties **12**

8 Estimation of CNC thickness using atomic force microscopy (AFM) **17**

9 Estimation of ensemble size values from individual particle sizes **21**

10 Correlating CNC phase behaviour with particle morphology **23**

11 Classification of CNC particles **23**

12 Supplementary Methods **27**
12.1 Dynamic light scattering (DLS) 27
12.2 Electrolytic conductivity and pH measurement 28
12.3 Optical spectroscopy of CNC photonic films 28
12.4 Scanning electron microscopy (SEM) of CNC photonic films 28
12.5 Conductometric titration of CNC suspensions 28
12.6 Zeta potential of CNC suspensions 28
12.7 UV-vis transmission spectroscopy of CNC suspensions 28
12.8 Cryogenic transmission electron microscopy (cryoTEM) of CNC suspensions 29
12.9 Atomic force microscopy (AFM) of CNC suspensions 29
12.10 Transmission electron microscopy (TEM) shape analysis 30

1 Calibration of sonication dose by calorimetry

The impact of ultrasonication of CNC suspensions depends on the properties of the suspension (volume and CNC concentration), the settings on the tip sonicator (tip amplitude) and the duration of the treatment (Supplementary Figure 1A). Although sonication is frequently applied to CNC suspensions and the equipment settings are often provided in the literature, it can be difficult to make quantitative comparisons between results. To explore the consistency of sonication across different experimental conditions, the hydrodynamic diameter of CNC particles after various sonication treatments was measured using DLS (Supplementary Methods, section 12.1). The different treatment conditions are summarised in Supplementary Figure 1B).

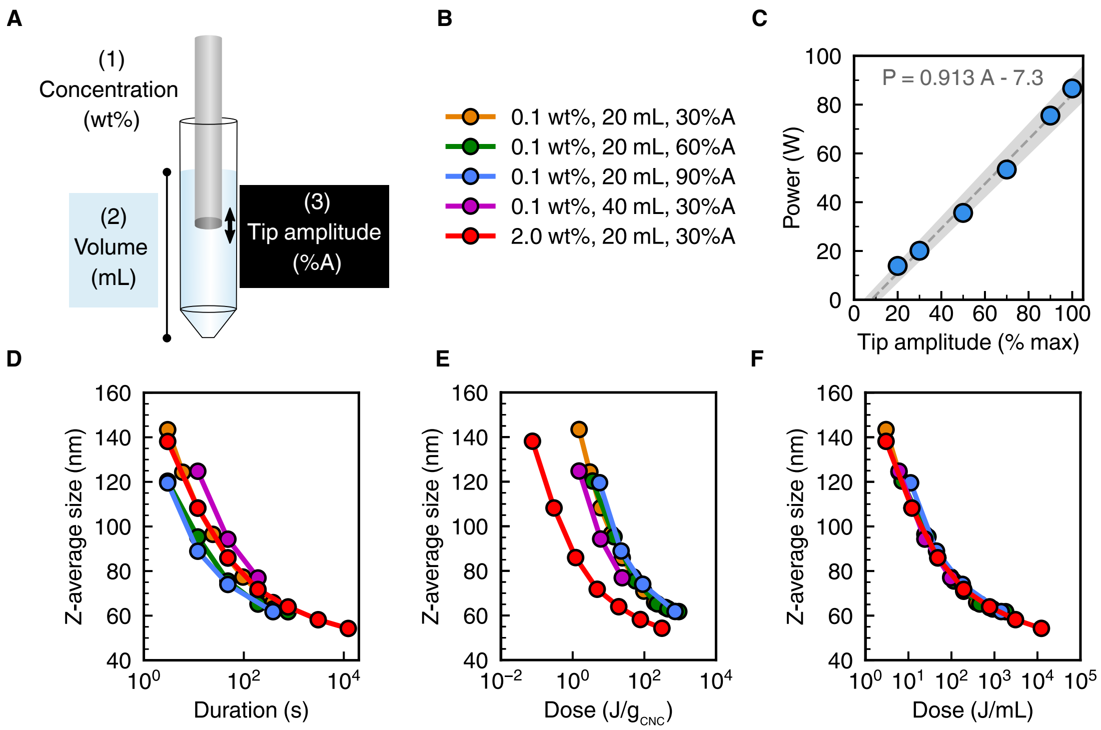

Supplementary Figure 1: (**A**) Schematic of experimental setup for tip sonication, with key experimental parameters indicated. (**B**) List of experimental conditions used for calibration experiments. Colour scheme matches that used in (**D**-**F**). (**C**) Caloric power delivered to suspension versus tip amplitude, with linear fitting. (**D**-**F**) DLS z-average size versus various dose parameters: (D) duration of treatment, (E) energy per CNC dry mass w_S, and (F) energy per suspension volume u_S.

To quantify the impact of sonication in a device-independent manner, it is necessary to express the sonication dose as the total energy delivered to the system by the sonicator tip. As sonication increases the temperature of the suspension, the energy delivered to the suspension can be estimated by calorimetry. Calorimetry was performed using a custom-made bomb calorimeter, consisting of a cylindrical chamber of expanded polystyrene foam surrounding a centrifuge tube to thermally insulate the sample. The temperature was measured using a thermocouple (Thorlabs TSP01) within the chamber. A magnetic stirrer bar was used to maintain mixing of the suspension.

First, the power P delivered to a sample of 40 g deionised water was determined for a range of values for the amplitude of the sonicator tip (expressed as a percentage of the maximum amplitude, $\%A$), as shown in Supplementary Figure 1C. Using this calibration curve for $P(\%A)$, the sonication energy E delivered to the sample is given by

$$E(\%A) = P(\%A)t \tag{1}$$

where t is the total duration of the sonication treatment (considering only the duration of the ON portion of the ON:OFF sonication cycles). This expression assumes that the CNC suspensions have the same specific heat capacity as water, which is a valid assumption at low CNC concentration.

The decrease in hydrodynamic diameter (DLS z-average size) with sonication is shown in Supplementary Figure 1D-F, where the sonication dose is expressed in terms of sonication duration t (Supplementary Figure 1D), energy per CNC dry mass ($w_S = E/m_{CNC}$, Supplementary Figure 1E) and energy per suspension volume ($u_S = E/V$, Supplementary Figure 1F).

It is clear from Supplementary Figure 1D-F that the hydrodynamic diameter results are only consistent across all experimental conditions when the sonication dose is expressed using u_S. Note that energy per CNC dry mass w_S, a dose unit often used in the CNC literature, gives consistent results when comparing conditions at fixed CNC concentration, but not when comparing doses at different concentrations (Supplementary Figure 1E).

2 Pitch measurement by SEM on CNC film cross-sections

The pitch of CNC photonic films can be directly observed from the film cross-section in SEM images (Supplementary Methods, section 12.4). The helicoidal structure of CNC photonic films results in a periodic texture in SEM cross-sections, as shown for selected samples in Supplementary Figure 2. The pitch is determined from the vertical distance for one full rotation of the structure (corresponding to two repeats of the fingerprint pattern). Cross-sectional SEM images for $u_S = 0\,\mathrm{J\,mL^{-1}}$ and $u_S > 2\times10^3\,\mathrm{J\,mL^{-1}}$ appeared isotropic with no helicoidal texture.

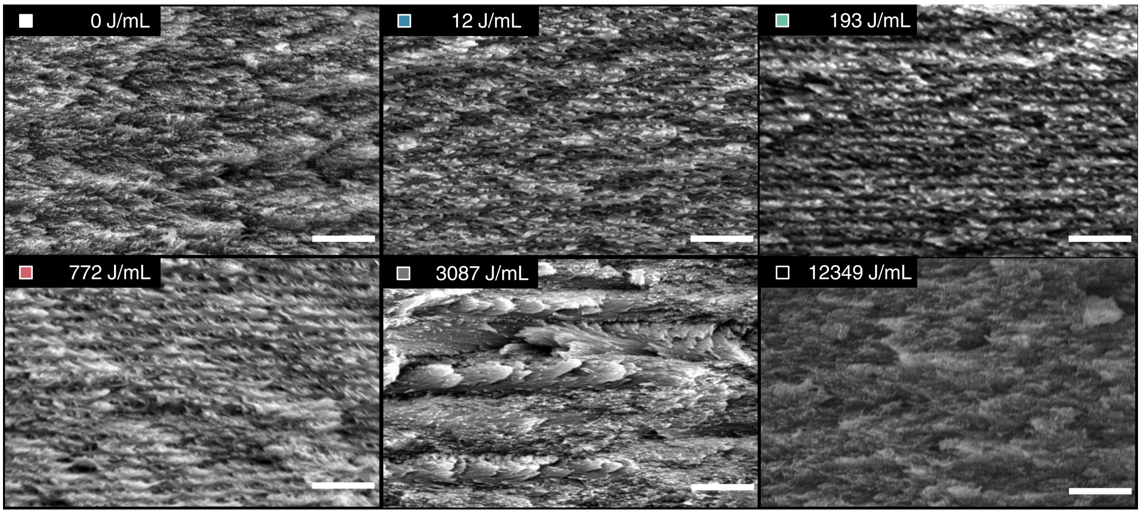

Supplementary Figure 2: Examples of SEM cross-sections for CNC photonic films. Dose indicated by the value on each image. Scale bar is 1 µm.

3 Pitch measurement by optical spectroscopy on CNC photonic films

The left-handed helicoidal configuration of birefringent CNCs in a solid film results in selective reflection of left-circular polarised (LCP) light in a wavelength range determined by the cholesteric pitch P. The peak reflection wavelength at normal incidence λ_{max} is given by

$$\lambda_{max} = n_{\mathrm{CNC}} P, \tag{2}$$

where n_{CNC} is the average refractive index of the CNC film, which we assumed to be 1.555 based on previous reports (1).

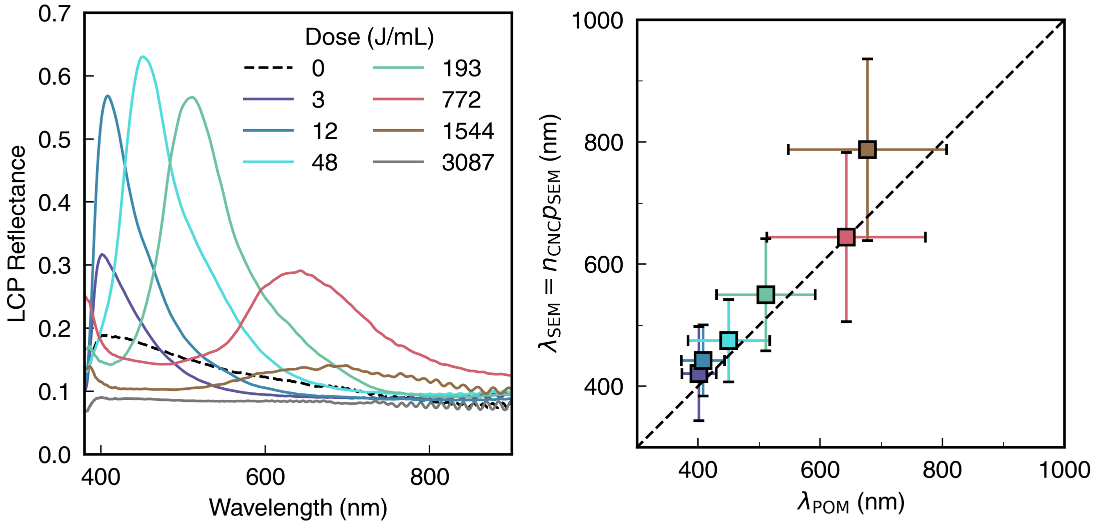

Supplementary Figure 3: (**A**) Left-circular polarised (LCP) reflectance spectra of CNC photonic films. (**B**) Comparison of peak LCP reflectance wavelength from polarised optical microscopy λ_{POM} and the predicted peak reflection wavelength based on SEM pitch values $\lambda_{\mathrm{SEM}} = n_{\mathrm{CNC}} P_{\mathrm{SEM}}$, assuming $n_{\mathrm{CNC}} = 1.555$. The error bars for λ_{POM} indicate the peak full width at half maximum, while the error bars for λ_{SEM} indicate the standard deviation of the pitch measurements.

Left-circular polarised reflection spectra were obtained at a range of sonication doses (Supplementary Methods, section 12.3). The peak reflection wavelength increases with sonication dose, as shown in Supplementary Figure 3. The apparent peak for the never-sonicated sample ($u_S = 0\,\mathrm{J\,mL^{-1}}$) could indicate the presence of some chiral nematic domains within the structure, although no ordering was visible in SEM images of the film cross-section (Supplementary Figure 2). This small peak could also be attributed to scattering in the disordered structure, or partial absorption by the reference silver mirror in this wavelength range. The peak reflection wavelength at high doses ($u_S > 2000\,\mathrm{J\,mL^{-1}}$) was too far into the infrared to be detected using this setup. There is reasonably good agreement between the peak LCP reflectance wavelength obtained by spectroscopy and the pitches measured by SEM (Supplementary Figure 2). Determination of CNC pitch by SEM tends to over-estimate the true value as the pitch of tilted chiral nematic domains appears larger when viewed from the plane of film breakage.

4 Discussion of other explanations of pitch increase induced by sonication

4.1 Release of trapped ions

The breakdown of CNCs is associated with the release of trapped ions, increasing the electrolytic conductivity of the suspension. The conductivity and pH of sonicated CNC suspensions were measured after sonication but before dialysis (Supplementary Methods, section 12.2), producing the results shown in Supplementary Table 1.

Sonication dose (J/mL)	Conductivity (μS cm^{-1})	pH
0	362.1 ± 0.2	2.76
12	428.0 ± 0.4	2.71
48	500.9 ± 1.0	2.67
193	802.0 ± 0.9	2.55
772	1535.9 ± 1.8	2.34

Supplementary Table 1: Table of electrolytic conductivity and pH for sonicated CNC suspensions before dialysis.

The decrease in pH with sonication indicates that H^+ ions are released, and the associated contribution to the conductivity increase can be estimated from the pH shift. Explicitly, denoting the conductivity and pH of the never-sonicated suspension as S_0 and pH_0 respectively, the expected conductivity contribution from H^+ ions is

$$\Delta S_{H^+} = \Lambda_{H^+}(10^{-\mathrm{pH}} - 10^{-\mathrm{pH}_0}) \tag{3}$$

where Λ_{H^+} ($\approx 0.035\ \mathrm{m^2\,S\,mol^{-1}}$) is the molar ionic conductivity of H^+ in aqueous solution, using the value at infinite dilution as an approximation (2). The estimated H^+ contribution compared to the experimental conductivity change is shown in Supplementary Figure 4. These results indicate that the release of H^+ ions is the dominant source of conductivity change. It is likely that the released H^+ ions originate from the acid hydrolysis used to produce the CNCs.

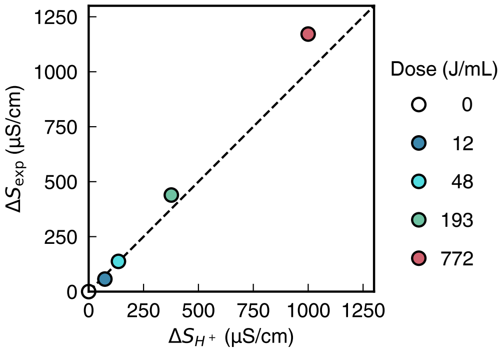

Supplementary Figure 4: Comparison of estimated H^+ contribution to the conductivity change calculated using Supplementary Table 1 and eq. (3) versus experimentally-measured conductivity change. Dotted black line indicates equality.

It has been proposed that these released ions increase the effective particle volume and thereby cause a red-shift in the chiral nematic pitch by preventing short-range chiral interactions between CNCs (3). However, this argument seems to contradict the expected behaviour of charged colloids, where increasing ionic strength is usually found to reduce the thickness of the electric double layer (4). Furthermore, numerous studies have shown that increasing the ionic strength of liquid crystalline CNC suspensions results in a smaller pitch, leading to a blue-shifted colour in the photonic film (5). In this work, the suspensions were therefore extensively dialysed (> 1 week) after sonication, all in the same bath of deionised water, to rule out the influence of released ions. After dialysis, an apparent difference in conductivity was measured between samples and could not simply be attributed to variation in CNC concentration after dialysis (Supplementary Table 2)

Sonication dose (J/mL)	Conductivity ($\mu S\,cm^{-1}$)	CNC concentration (wt%)
0	192.5 ± 2.0	1.99
12	262.4 ± 0.8	2.03
48	254.8 ± 1.8	1.80
193	343.2 ± 7.2	1.88
772	380.3 ± 2.8	1.93

Supplementary Table 2: Table of electrolytic conductivity and CNC concentration values for sonicated CNC suspensions after dialysis.

The simplest explanation of the higher conductivity values for sonicated suspensions after dialysis in Supplementary table 2 is that these suspensions contain more ions due to incomplete dialysis. Highly sonicated suspensions were also found to have lower pH values (data not shown), but pH values on low ionic strength suspensions are prone to error due to the diffusion of ions from the pH probe into the suspension. To more accurately quantify the suspension pH, we followed a recently reported procedure by preparing samples at fixed CNC concentration (1.50 wt%) with an excess of K^+ ions (50 mM KCl) to dislodge the H^+ ions from the CNC surface (6). Under these conditions, all five CNC suspensions were found to have the same pH value within experimental uncertainty (2.56±0.03), demonstrating that the overall number of H^+ counterions per CNC mass was identical. This result is in agreement with surface charge values determined by conductometric titration (section 4.2).

These results suggest that the apparently higher conductivity and lower pH for sonicated suspensions after dialysis are not due to incomplete dialysis, but can instead be attributed to increased counterion mobility after sonication. Tight binding of counterions to the CNC surface greatly reduces their effective mobility, and therefore their contribution to conductivity and pH measurements of dialysed CNC suspensions. This binding is weakened by sonication as the total CNC surface area increases (Supplementary Figure 10C), leading to higher conductivity values.

4.2 Surface charge and colloidal stability

The surface charge on CNCs influences their colloidal stability and self-assembly by modifying the electrostatic interactions between particles (7). The effect of sonication on the CNC

surface charge was therefore investigated as a possible source of the observed variation in the chiral nematic pitch.

The CNC surface charge (expressed as moles of counterions per kilogram of CNC dry mass) was determined by conductometric titration (Supplementary Methods, section 12.5) after dialysis. As shown in Supplementary Table 3 the surface charge per CNC dry mass did not vary with sonication dose within the uncertainty of the fitting of the titration curve. The surface charge for these samples ($\approx$ 153 mmol kg^{-1}) corresponds to a CNC sulphur content of 0.49% (w/w), or a degree of substitution of 2.5% on glucose monomers.

Although the CNC specific surface charge (i.e. surface charge per mass) S is readily accessible by experiment, a more relevant physical properties is charge per surface area σ, also known as the areal surface charge density, which is given by $\sigma = S/\mathrm{SSA}$, where SSA is specific surface charge (surface area per CNC mass). The SSA for a CNC particle of surface area Σ and volume V is given by $\mathrm{SSA} = \Sigma/(\rho_{\mathrm{CNC}} V)$, where ρ_{CNC} is the CNC mass density and assumed to be 1600 kg m^{-3}. For a distribution of particles, the mean surface charge density can be estimated by assuming the specific surface charge is identical for all particles, i.e.

$$\langle \sigma \rangle = \langle \frac{S}{\mathrm{SSA}} \rangle$$

as opposed to assuming the charge per surface area is identical (which would give $\langle \sigma \rangle' = S/\langle \mathrm{SSA} \rangle$). The CNC surface charge density value is therefore highly dependent on the method used to estimate SSA.

Estimates of the CNC charge per surface area obtained by three possible methods are shown in Supplementary Figure 5. The "TEM outline" estimate is obtained by calculating the average SSA using the SSA of each particle based on the surface area and volume estimates used elsewhere in this work (see section 7 and Supplementary Figure 10C). The "box" estimate is obtained in a similar way, but assumes that the CNCs are cuboidal, with length, width and thickness given by their box length L_b, box width W_b and mean thickness $\langle T \rangle$ respectively (see section 7 for definitions of these properties). The box estimate consistently produces a higher value for $\langle \sigma \rangle$, due to higher estimates of the particle volume. Alternatively, as shown in previous works (e.g. (7)), the CNC surface charge density can be estimated by assuming the CNCs are identical cylinders with length and diameter given by the box length and thickness. Using this "mean cylinder" approach, substantially lower estimates for $\langle \sigma \rangle$ were obtained. These results illustrate the considerable uncertainty in estimating the surface charge density of CNCs. Nevertheless, all estimates predict a decrease in $\langle \sigma \rangle$ with sonication dose

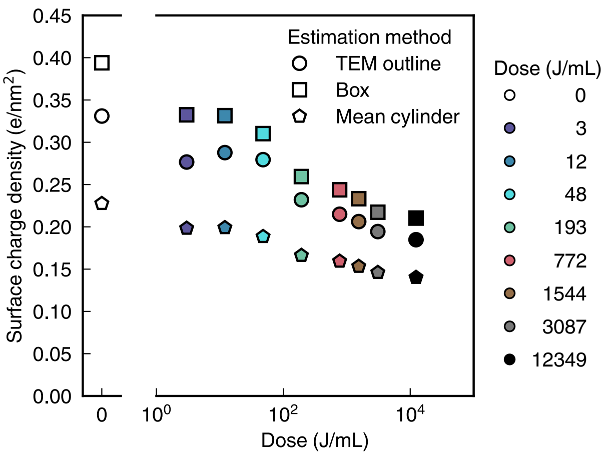

Supplementary Figure 5: Estimates of the CNC surface charge density versus sonication dose. Estimates are based on true particle shape (circles), box properties (squares) or by assuming all CNCs are cylinders with identical length and diameter (pentagons). Estimation methods are described further in the text.

The decrease in CNC surface charge density shown in Supplementary Figure 5 may be expected to indicate a decrease in colloidal stability. However, a complementary measurement of the mean electrophoretic mobility of the particles was performed by measuring the zeta potential (Supplementary Methods, section 12.6), which showed no clear trend with sonication dose within the uncertainty of the measurements. This result suggests that the expected decrease in charge per surface area does not make the CNCs colloidally unstable, at least under the conditions of the zeta potential measurement (ionic strength $I \approx 1$ mM). However, the decrease in $\langle \sigma \rangle$ with sonication may explain the earlier onset of kinetic arrest at higher doses.

Sonication dose (J/mL)	Specific surface charge (mmol/kg)	Zeta potential (mV)
0	154.1	-37.1 ± 7.5
12	149.9	-42.0 ± 10.6
48	155.0	-40.6 ± 10.7
193	154.2	-41.0 ± 11.7
772	154.3	-42.8 ± 11.4
3087	152.7	-41.0 ± 10.8

Supplementary Table 3: Table of colloidal properties of CNC suspensions.

5 Estimation of particle cross-section using turbidity spectra

Sonication visibly reduces the turbidity (*i.e.* cloudiness) of CNC suspensions due to the breakdown of scattering particles. The decrease in turbidity with sonication dose was determined by UV-vis transmission spectroscopy (Supplementary Methods, section 12.7) and is shown in Supplementary Figure 6.

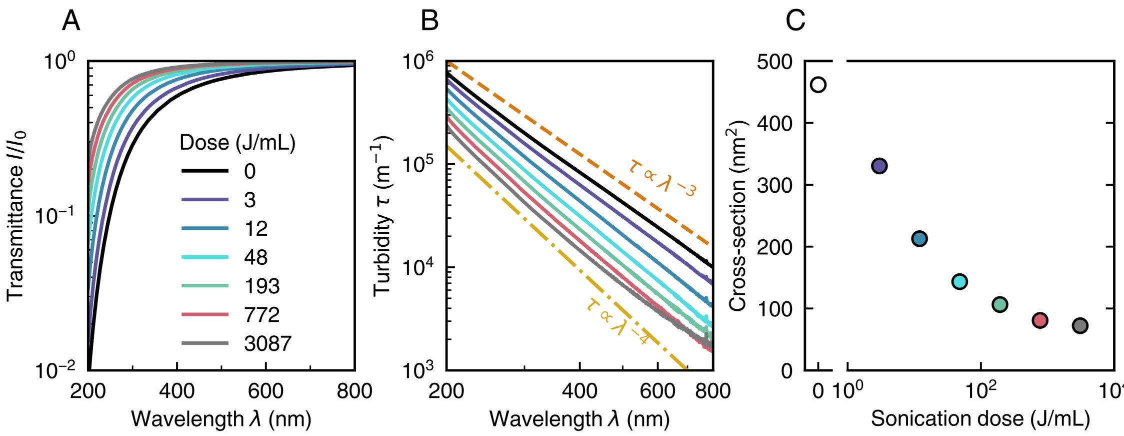

Supplementary Figure 6: (**A**) Transmission through 0.1 wt% CNC suspensions increases with sonication dose (**B**) The power-law scaling of the turbidity spectra is clearer in a log-log plot (**C**) Volume-weighted mean particle cross-section versus sonication dose.

In a log-log plot of turbidity versus wavelength (Supplementary Figure 6b), it is clear that alongside an overall decrease in turbidity at all wavelengths, sonication changes the shape of the turbidity spectrum, with a λ^{-3} wavelength dependence at low sonication dose transforming into a λ^{-4} dependence at high dose. This behaviour can be understood in terms of the morphological changes induced by sonication, and can be used to estimate the mean CNC particle cross-section (8).

For a turbid suspension of scattering dielectric particles in the limit of infinite dilution, light transmission obeys a Beer-Lambert-type decay with optical path length z:

$$I(z) = I_0 \exp(-kz). \tag{4}$$

For a suspension of identical particles p, the turbidity is given by $\tau = k/c_p$, where k is the decay constant in Equation (4) above and c_p is the particle concentration expressed as the number of particles per volume. If the particles are small compared to the wavelength ($v_p^{-1/3} < \lambda$) and have mild refractive index contrast with the solvent medium ($(n_p - n_0)/n_0 \ll 1$), the turbidity can be approximated using the Rayleigh-Gans-Debye model:

$$\tau_p = 24\pi^3 \frac{n_0^4 v_p^2}{\lambda^4} \left(\frac{n_p^2 - n_0^2}{n_p^2 + 2n_0^2} \right)^2 Q_p, \tag{5}$$

where Q_p is a geometric factor that depends only on the particle shape.

The turbidity of CNC suspensions can be understood by comparing two ideal shapes: isotropic spheres and long slender rods. For spherical particles, $Q_{sph} = 1$ and the turbidity is

$$\tau_{sph}(\lambda) = 24\pi^3 \frac{n_0^4 v_p^2}{\lambda^4} \left(\frac{n_p^2 - n_0^2}{n_p^2 + 2n_0^2} \right)^2 , \tag{6}$$

which is the conventional expression for Rayleigh scattering with λ^{-4} wavelength dependence. For a slender rod with an extended length $L_p \gg \lambda$ but sub-wavelength cross-section, the geometric factor is (9; 10)

$$Q_{rod} = \frac{11}{20} \frac{\lambda}{n_0 L_p}. \tag{7}$$

and the turbidity is therefore

$$\tau_{rod}(\lambda) = \frac{66\pi^3}{5}\frac{n_0^3}{\lambda^3}\frac{v_p^2}{L_p}\left(\frac{n_p^2 - n_0^2}{n_p^2 + 2n_0^2}\right)^2, \tag{8}$$

which has a λ^{-3} wavelength dependence. At low sonication dose, CNC particles behave like slender rods and therefore exhibit a λ^{-3} wavelength dependence. As sonication breaks apart the CNCs, reducing the particle volume and aspect ratio, their morphology becomes less elongated and a λ^{-4} wavelength dependence emerges (as seen in Supplementary Figure 6B).

The turbidity equations given by eq. (5) are difficult to apply directly to CNC suspensions because the number density of CNCs (c_p) cannot be directly measured, and there is considerable polydispersity in CNC size and shape. Therefore, we consider CNC suspensions as a population of different particle species p, each with different particle volumes v_p but identical refractive indices $n_p = n_1$, suspended in a medium of index n_0. In this case, the turbidity decay constant in eq. (4) generalises to

$$k = \sum_p \tau_p c_p = 24\pi^3\frac{n_0^4}{\lambda^4}\left(\frac{n_1^2 - n_0^2}{n_1^2 + 2n_0^2}\right)^2 \sum_p Q_p v_p^2 c_p \tag{9}$$

where $\sum_p$ indicates summation over all particle species. Experimentally, the most accessible concentration metric is the overall particle volume fraction, given by

$$\phi = \sum_p v_p c_p = \sum \phi_p. \tag{10}$$

We therefore define the turbidity per volume fraction $\tau' = k/\phi$, or explicitly

$$\tau' = 24\pi^3\frac{n_0^4}{\lambda^4}\left(\frac{n_1^2 - n_0^2}{n_1^2 + 2n_0^2}\right)^2 \frac{\sum_p Q_p v_p^2 c_p}{\sum_p v_p c_p} \tag{11}$$

For a distribution of slender rods (with Q_p given by Equation (7) for all particle species), we find that

$$\tau' = \frac{66\pi^3}{5}\frac{n_0^3}{\lambda^3}\left(\frac{n_1^2 - n_0^2}{n_1^2 + 2n_0^2}\right)^2 \langle A\rangle \tag{12}$$

where $\langle A\rangle$ is the volume-weighted mean of the particle cross-section:

$$\langle A\rangle = \frac{\sum_p (v_p^2/L_p) c_p}{\sum_p v_p c_p} = \frac{\sum_p (v_p/L_p)\phi_p}{\sum_p \phi_p} \tag{13}$$

Equation (12) can therefore be used to estimate the particle cross-section from the experimental transmission spectra (Supplementary Figure 6C), a technique that has previously been reported for CNC suspensions (8). Only the long-wavelength data ($\lambda > 400$ nm) were used, as the assumptions used to derive Equation (12) are only valid in the long-wavelength limit. The mean cross-section $\langle A\rangle$ decreases with sonication dose (Supplementary Figure 6c) and approaches a limiting value of $\approx 70\,\text{nm}^2$ at high sonication dose.

6 Evidence of bundles from cryoTEM imaging

The preparation of CNC samples for conventional TEM imaging involves drying a droplet of sample onto the TEM grid, which can cause aggregates to appear in TEM images that do not exist in the original suspension. These artefacts should not be confused with CNC bundles, which are native to CNC suspensions at all sonication doses, or with large clusters of bundles at low sonication doses (classified as aggregates in the article). To illustrate that bundles are a native feature of CNC suspensions, cryoTEM imaging was performed on selected CNC samples (Supplementary Methods, section 12.8). In cryoTEM imaging, the CNC suspension is frozen and never dried, thus eliminating any risk of artefact creation. Example images are shown in Supplementary Figure 7. It is evident from qualitative inspection of the images that these suspensions contain CNCs with a bundled morphology.

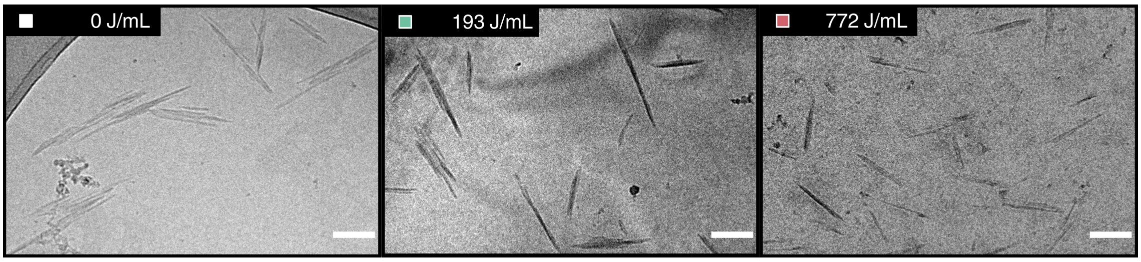

Supplementary Figure 7: Examples of cryoTEM images for CNC suspension, illustrating the presence of bundles. Sonication dose u_S is indicated by the value on each each image. Scale bar is 200 nm.

7 Definitions and distributions of morphological properties

The morphological properties used in this work are summarised in Supplementary Tables 4 to 6. The corresponding distributions for each sonication dose are Supplementary Figures 8 to 10

Property	Symbol	Equation	Units
Area, true shape	A		nm^2
Area, convex hull	A_{CH}		nm^2
Area, oriented bounding box	A_b	$L_b W_b$	nm^2
Perimeter, true shape	P		nm
Perimeter, convex hull	P_{CH}		nm
Length, oriented bounding box	L_b		nm
Length, Feret (max. caliper length)	L_F		nm
Width, oriented bounding box	W_b		nm
Width, area-equivalent (AE)	W_{AE}	$A/L_b = \mathcal{R} W_b$	nm
Width, Feret (min. caliper length)	W_F		nm

Supplementary Table 4: Table of size properties measured from TEM images, with definitions. The distributions for L_b and W_{AE} are given in the main text; the distributions for other properties are plotted in Supplementary Figure 8.

Property	Symbol	Equation	Units
Aspect ratio, oriented bounding box	α_b	L_b/W_b	-
Aspect ratio, area-equivalent (AE)	α_{AE}	$L_b/W_{AE} = L_b^2/A$	-
Isoperimetric Quotient, 2D (Thinness Ratio)	IQ_2	$4\pi A/P^2$	-
Solidity	$\mathcal{S}$	A/A_{CH}	-
Convexity	$\mathcal{C}$	P/P_{CH}	-
Rectangularity	$\mathcal{R}$	A/A_b	-
Perimetric rectangularity	$\mathcal{R}_P$	$P/2(L_b + W_b)$	-

Supplementary Table 5: Table of shape properties measured from TEM images, with definitions. Distributions for $\mathcal{R}$ are given in the main text; the distributions for other properties are plotted in Supplementary Figure 9.

Property	Symbol	Equation	Units
Thickness, particle mean	$\langle T \rangle$		nm
Area, particle surface	Σ	$2A + P\langle T \rangle$	nm^2
Volume	V	$A\langle T \rangle$	nm^3
Specific surface area	–	$\Sigma/(\rho_{CNC} V)$	m^2/g
Isoperimetric Quotient, 3D	IQ_3	$36\pi V^2/\Sigma^3$	-
Circle-equivalent (CE) diameter	D_{CE}	$\sqrt{4 W_{AE}\langle T \rangle/\pi}$	-
Aspect ratio, 3D	α_{3D}	$L_b/\sqrt{W_{AE}\langle T \rangle}$	nm
Cross-section (XS) aspect ratio, area equivalent	$\alpha_{XS,AE}$	$W_{AE}/\langle T \rangle$	-
Cross-section (XS) aspect ratio, box	$\alpha_{XS,b}$	$W_b/\langle T \rangle$	-
Max-min (MM) aspect ratio, box	α_{MM}	$L_b/\langle T \rangle$	-

Supplementary Table 6: Table of size and shape properties estimated using AFM and TEM data, with definitions. Distributions for $\langle T \rangle$ are plotted in Supplementary Figure 12. Distributions for α_{3D} are given in the main text. Distributions for other properties are plotted in Supplementary Figure 10.

u_S (J/mL)	# particles, TEM
0	254
3	258
12	257
48	263
193	256
772	396
1544	506
3087	485
12349	540

Supplementary Table 7: Table of counts of CNC particles at each sonication dose measured in TEM morphological analysis.

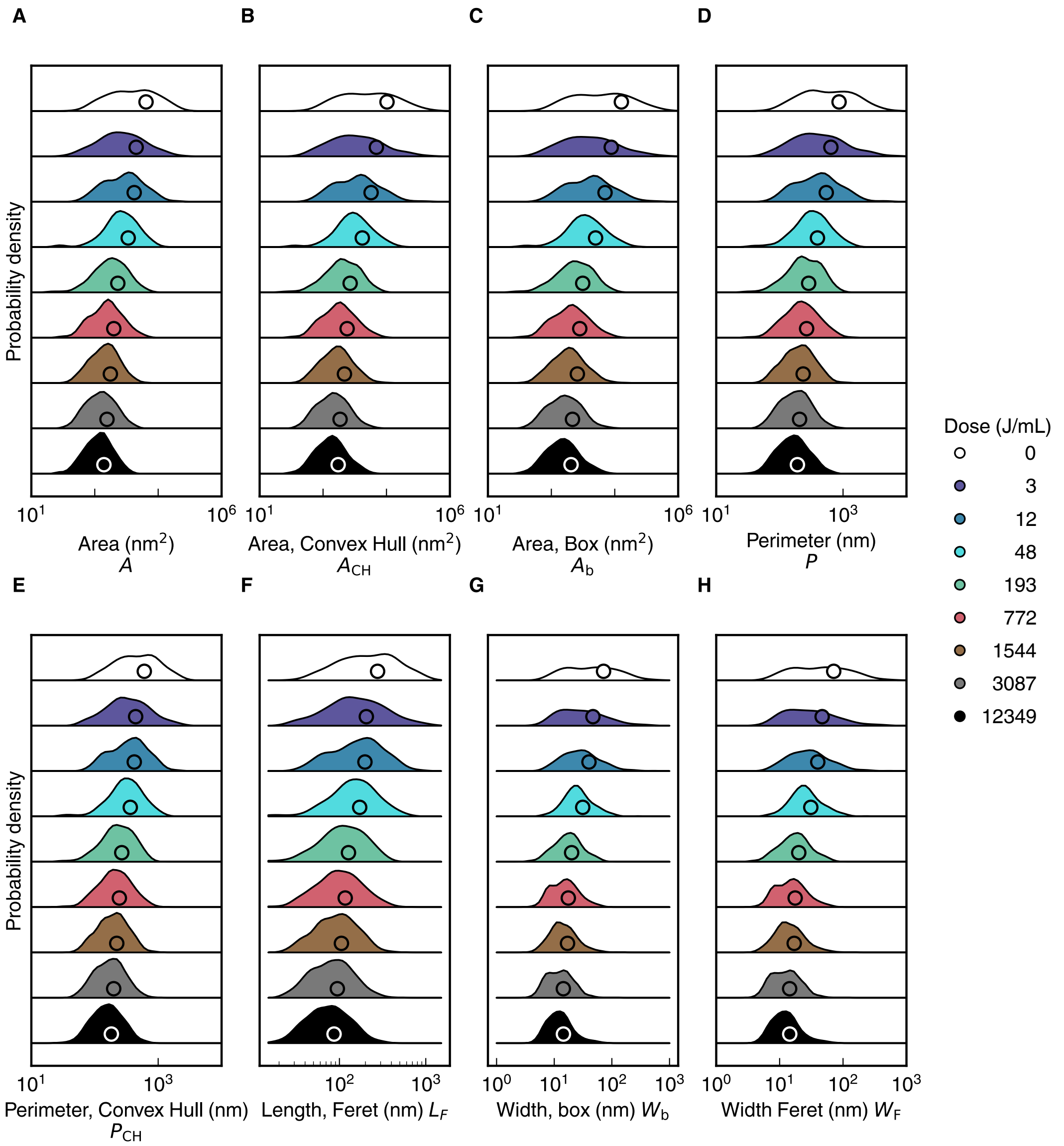

Supplementary Figure 8: Size distributions for additional properties: (**A**) Area A (**B**) Area of convex hull A_{CH} (**C**) Area of oriented bounding box A_{b} (**D**) Perimeter P (**E**) Perimeter of convex hull P_{CH} (**F**) Feret length L_F (**G**) Width of oriented bounding box W_{b} and (**H**) Feret width (minimum caliper length) W_F The properties are defined in Supplementary Table 5.

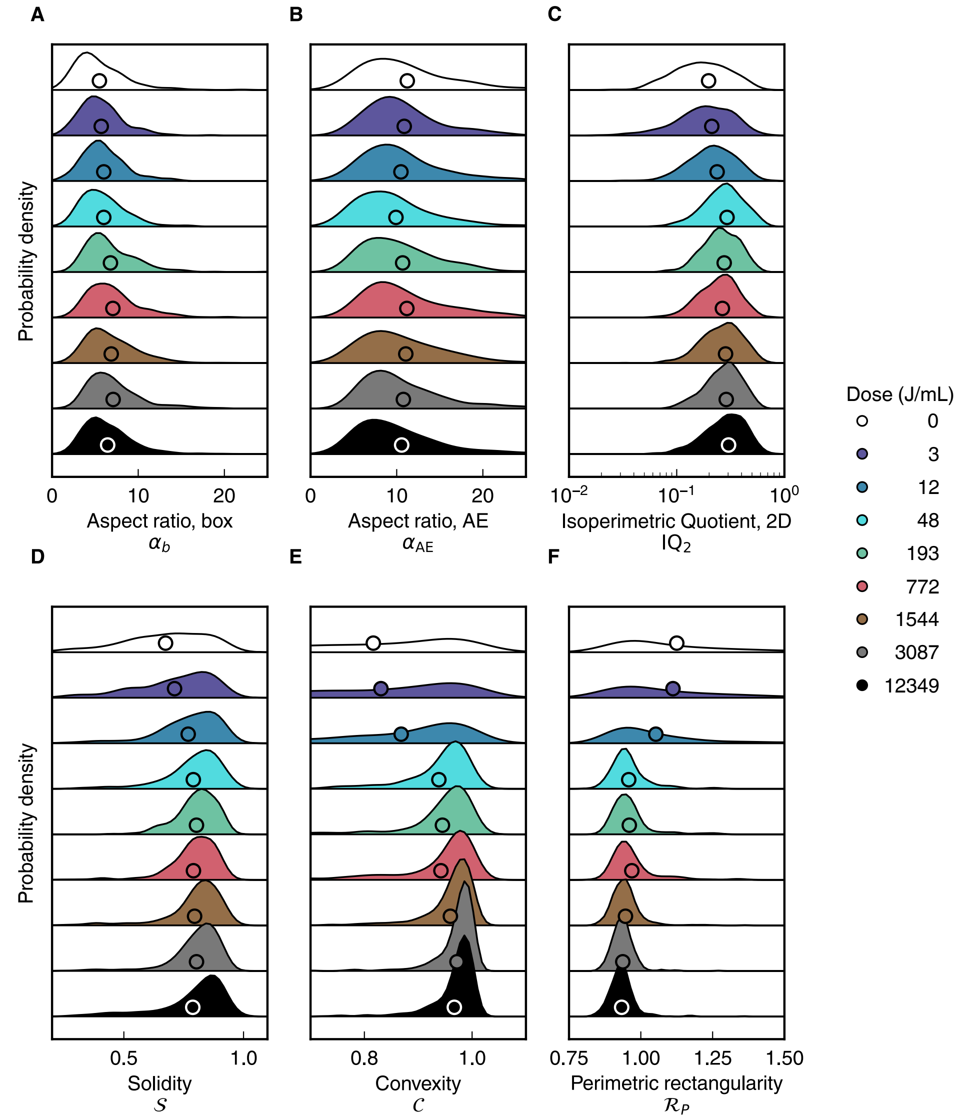

Supplementary Figure 9: Shape distributions for additional properties: (**A**) Aspect ratio of oriented bounding box α_b (**B**) Aspect ratio using box length and area-equivalent width α_{AE} (**C**) 2D Isoperimetric quotient IQ_2 (**D**) Solidity $\mathcal{S}$ (**E**) Convexity $\mathcal{C}$ (**F**) Perimetric rectangularity $\mathcal{R}_P$.The properties are defined in Supplementary Table 5.

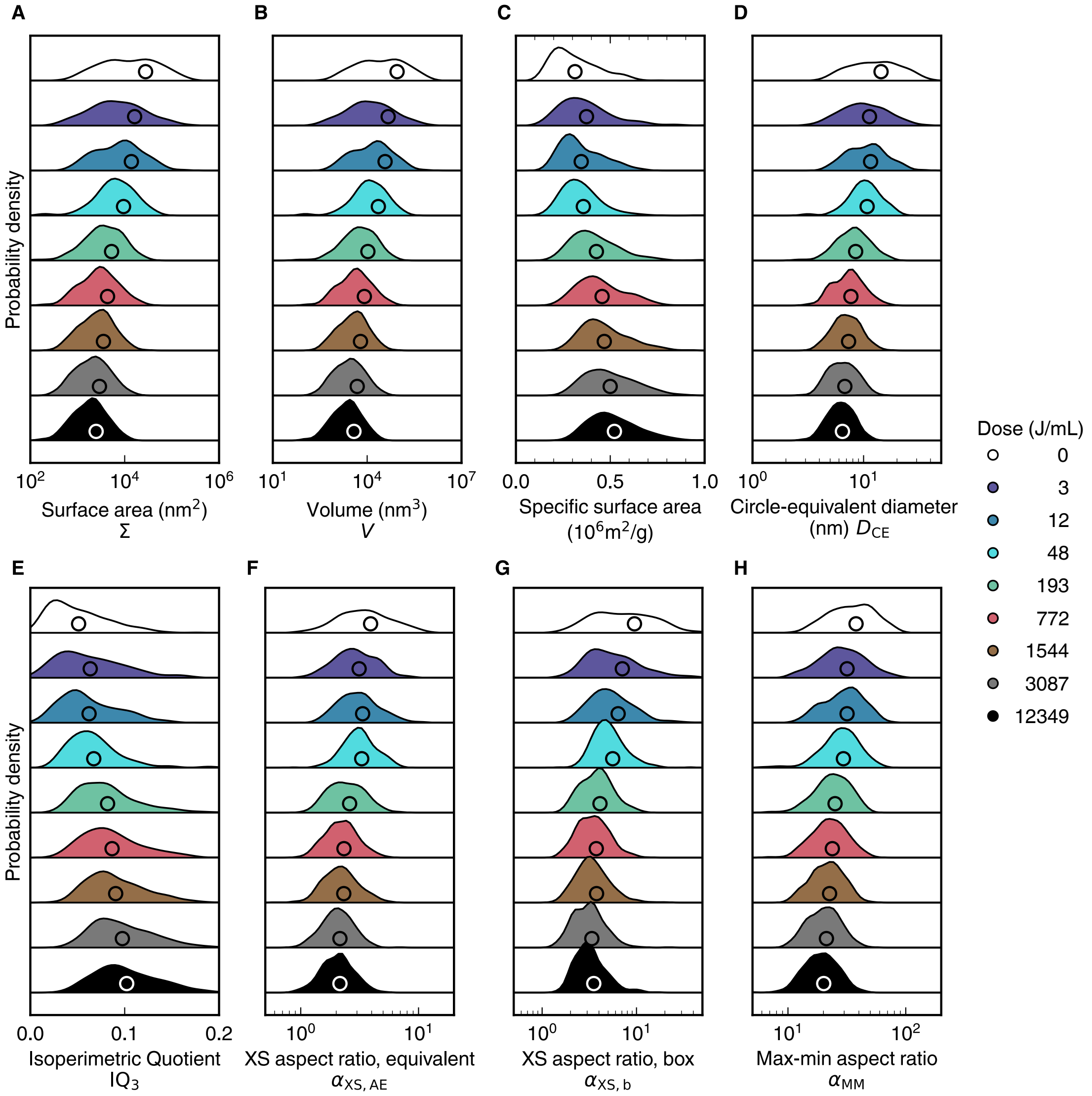

Supplementary Figure 10: Shape distributions for additional 3D morphological properties: (**A**) Surface area Σ (**B**) Volume V (**C**) Specific surface area (**D**) circle-equivalent diameter D_{CE} (**E**) 3D Isoperimetric quotient IQ_3 (**F**) Cross-sectional aspect ratio using AE width $\alpha_{\mathrm{XS,AE}}$ (**G**) Cross-sectional aspect ratio using box width $\alpha_{\mathrm{XS,b}}$ (**H**) Max-min aspect ratio α_{MM}. These properties are defined in Supplementary Table 6.

8 Estimation of CNC thickness using atomic force microscopy (AFM)

Atomic force microscopy (AFM) provides data on the topography of a sample surface, and therefore offers complementary morphological information to transmission electron microscopy (TEM). For CNCs deposited on a flat substrate and imaged using AFM, the mean and maximum thickness of the particle can be accurately measured, as illustrated in Supplementary Figure 11A. The lateral size of particles, as expressed as the Feret length and width (Supplementary Figure 11B), can also be obtained. However, it should be noted that the XY topography of the particle is convolved by the effective diameter of the AFM tip (Supplementary

Figure 11C), which limits the use of AFM data for more detailed morphological analysis.

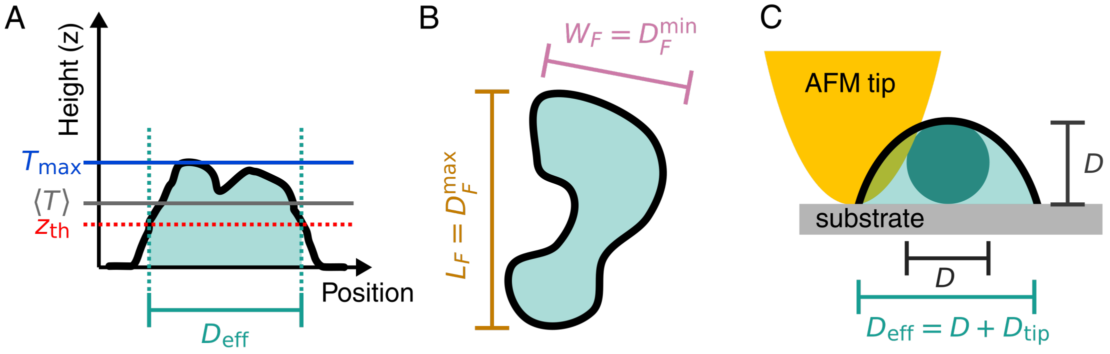

Supplementary Figure 11: Morphological properties obtained from atomic force microscopy (AFM). (**A**) Individual particles (also known as "grains" in AFM terminology) are identified as the region where the baseline-corrected surface height $z(x, y)$ exceeds a critical threshold value z_{th} (red dotted line). Each particle is defined as the bounded region that satisfies the threshold criterion $z \geq z_{th}$. A threshold of z_{th} = 1.2 nm was used in this work. The mean thickness $\langle T \rangle$ of each particle (grey line) is obtained by averaging the height value within the particle boundary. The maximum thickness T_{max} (blue line) can also be obtained. (**B**) The Feret (caliper) diameter of a particle in the substrate plane, D_F, is defined as the smallest distance between oriented parallel lines that encloses the particle. The value of D_F depends on the orientation of the parallel lines relative to the particle. The Feret length L_F (orange) and Feret width W_F (pink) of the particle are defined as the maximum and minimum Feret diameter respectively. Note that the parallel lines that enclose L_F and W_F are not at perpendicular orientations (unlike the definition of the box length L_b and box width W_b, Supplementary Table 5). (**C**) Illustration of the effect of a tip artefact on a spherical nanoparticle (green) on a flat substrate. For a spherical nanoparticle, the bare particle diameter D is accurately measured in the height profile but not in the lateral scan direction due to the width of the AFM tip D_{tip}, leading to an effective diameter D_{eff} being measured.

CNCs at a range of sonication doses were imaged and analysed using AFM (see Supplementary Methods section 12.9 and Supplementary Table 8 for the number of particles measured for each dose). This analysis shows that sonication reduces the thickness of CNC particles (Supplementary Figure 12), as well as the length and width (Supplementary Figure 13).

u_S (J/mL)	# particles, AFM
0	90
12	302
193	369
772	554
12349	1344

Supplementary Table 8: Table of counts of CNC particles at a range of sonication doses measured in AFM morphological analysis.

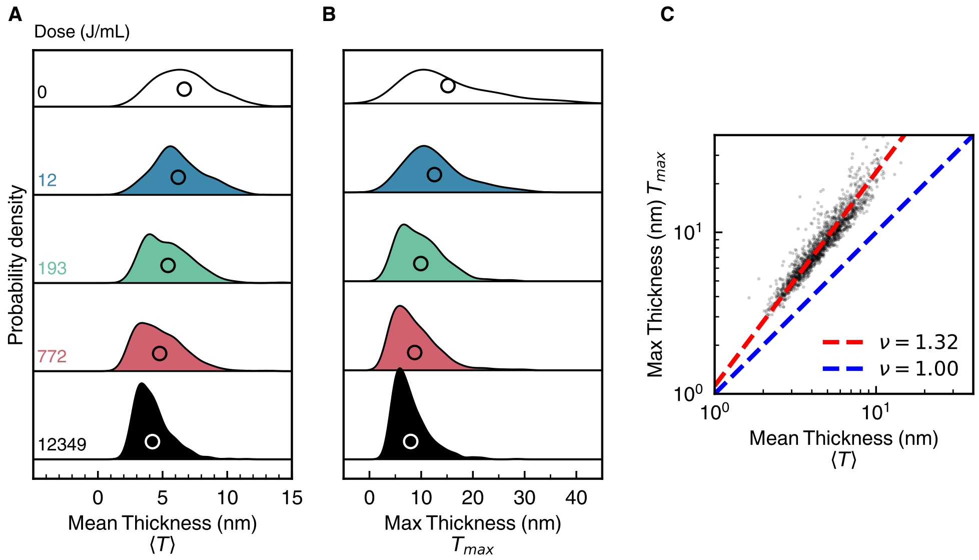

Supplementary Figure 12: Statistics for CNC thickness from AFM image analysis. Size distributions for mean thickness $\langle T \rangle$ (**A**) and max thickness T_{max} (**B**), demonstrating the reduction in thickness with sonication dose limited by the minimum thickness of a single cellulose Iβ crystallite ($\approx$ 3 nm). (**C**) 2D scatter plot of mean versus max thickness, combining data for all sonication doses. Remarkably, a power law relation between mean and max thickness ($T_{\text{max}} \propto \langle T \rangle^{\gamma}$) is well-described by an exponent $\nu = 1.32 \approx 4/3$ (red dotted line), rather than a linear fitting ($\nu = 1$, blue dotted line, shifted vertically for clarity). This scaling behaviour can be attributed to the bundled, fractal-like 3D morphology of the CNC particles. It should be noted that different particle populations (Aggregates, Bundles, Crystallites and Distorted crystallites) are expected to show different scaling behaviour, but the classification used for particle shapes from TEM images could not be directly applied to AFM shape data.

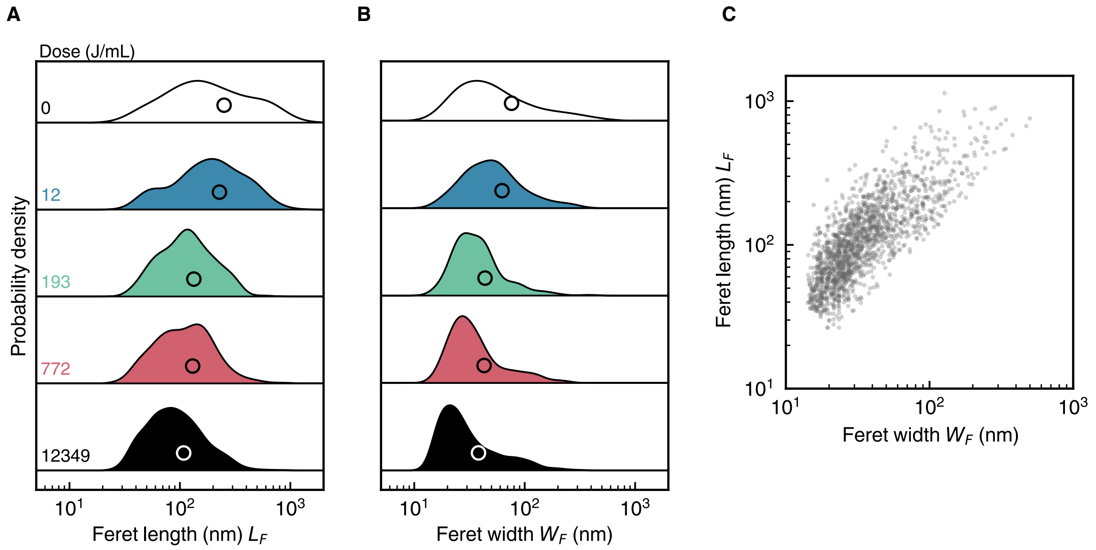

Supplementary Figure 13: Statistics for Feret length and width from AFM image analysis. Size distributions for Feret length L_F (**A**) and Feret width W_F (**B**), demonstrating the reduction in overall particle size with increasing sonication dose. Circles indicate mean values. (**C**) 2D scatter plot of Feret width versus Feret length, combining data for all sonication doses.

AFM data can also be used to estimate the thickness of a given CNC from a TEM image by matching the particle length to a length-thickness calibration curve obtained from AFM image analysis. As shown in Supplementary Figure 14A, the AFM Feret length shows a clear positive correlation with mean thickness, which can be modelled by an empirical power law relation. Length values from AFM and TEM show fairly good agreement, even without correcting for the tip diameter (Supplementary Figure 14B). Consequently, the thickness of particles measured from TEM images was estimated by assuming $L_f^{\text{TEM}} = L_f^{\text{AFM}}$ and applying the power law fitting from Supplementary Figure 14A. These thickness values were then used to estimate other shape properties (e.g. the 3D aspect ratio $\alpha_{3\text{D}}$).

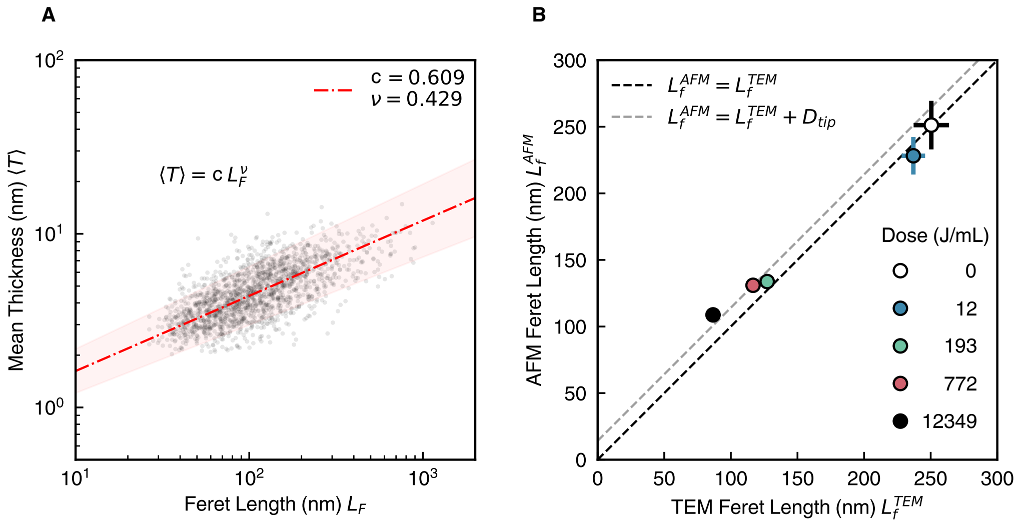

Supplementary Figure 14: (**A**) Correlation between Feret length L_F and mean thickness $\langle T \rangle$ from AFM image analysis. Red line indicates power law fitting $\langle T \rangle = cL_F^\nu$. Red shaded area indicates uncertainty. (**B**) Comparison of mean Feret length obtained from TEM analysis (L_f^{TEM}) versus AFM (L_f^{AFM}). Error bars indicate standard error of the mean. Good agreement is obtained between TEM and AFM values when adding a correction for the tip diameter (grey dotted line), especially at high sonication doses where the error bars are smaller.

9 Estimation of ensemble size values from individual particle sizes

The size distributions obtained from TEM images can be used to estimate ensemble properties such as the mean particle cross-section from UV-vis transmission spectroscopy (Supplementary Methods, section 12.7, section 5) or the mean hydrodynamic diameter obtained from DLS measurements (Supplementary Methods, section 12.1). The mean particle cross-section was estimated using eq. (13) in section 5, while the hydrodynamic diameter was estimated by the method explained below.

The mean hydrodynamic diameter $\langle d_h \rangle$ obtained from a DLS measurement is the hydrodynamic diameter corresponding to the average translational diffusion coefficient $\langle D_t \rangle$:

$$\langle d_h \rangle = \frac{k_B T}{3\pi \eta_0 \langle D_t \rangle} \tag{14}$$

where k_B is the Boltzmann constant, T is temperature, η_0 is the suspension viscosity (assumed to be the viscosity of water).

The average diffusion coefficient $\langle D_t \rangle$ is weighted by the scattered light intensity, which scales with the particle volume squared. The particle sizes measured from TEM can be used to estimate the intensity-averaged diffusion coefficient:

$$\langle D_t \rangle = \frac{\sum_p v_p^2 D_t}{\sum_p v_p^2} \tag{15}$$

where v_p is the particle volume and $\sum_p$ indicates summation of all the particles measured. The translation diffusion coefficient of CNCs can be estimated by assuming CNCs diffuse like ideal rods. The diffusion coefficient for an ideal rod of length L and aspect ratio α is (11):

$$D_t = \frac{k_B T}{3\pi\eta_0}\frac{\ln\alpha + \nu(\alpha)}{L} \tag{16}$$

where $\nu(\alpha)$ is a correction added to account for end effects:

$$\nu(\alpha) \approx 0.312 + 0.565\frac{1}{\alpha} - 0.100\frac{1}{\alpha^2}. \tag{17}$$

To apply these equations to CNC suspensions, the particle length was assumed to be the box length ($L = L_b$) and the aspect ratio was assumed to be the 3D aspect ratio ($\alpha = \alpha_{3D}$). Note that these values do not take into account electroviscous effects due to the electric double layer around the particles in aqueous suspension. For the CNC suspensions used for DLS measurements in this work, the hydrodynamic diameter is d ~100 nm and the Debye length κ^{-1} ~ 10 nm: in this limit (κd ~ 10), electroviscous effects are expected to be negligible (<5%), and are therefore not taken into account (12).

The estimated z-average hydrodynamic diameter calculated using Equations (14) to (17) is shown in Supplementary Figure 15A alongside the experimental values from DLS. The particle cross-section was estimated using eq. (13) and is shown in Supplementary Figure 15B alongside values obtained from UV-vis transmission spectroscopy (Section 5). The estimated values capture the overall trends and show good agreement, especially at higher sonication doses. The discrepancies at low sonication dose can be attributed to the irregular shapes of large particles, which deviate from the cylinder model, and also the sensitivity of the scattering intensity to fluctuations in the number of large particles observed in TEM images.

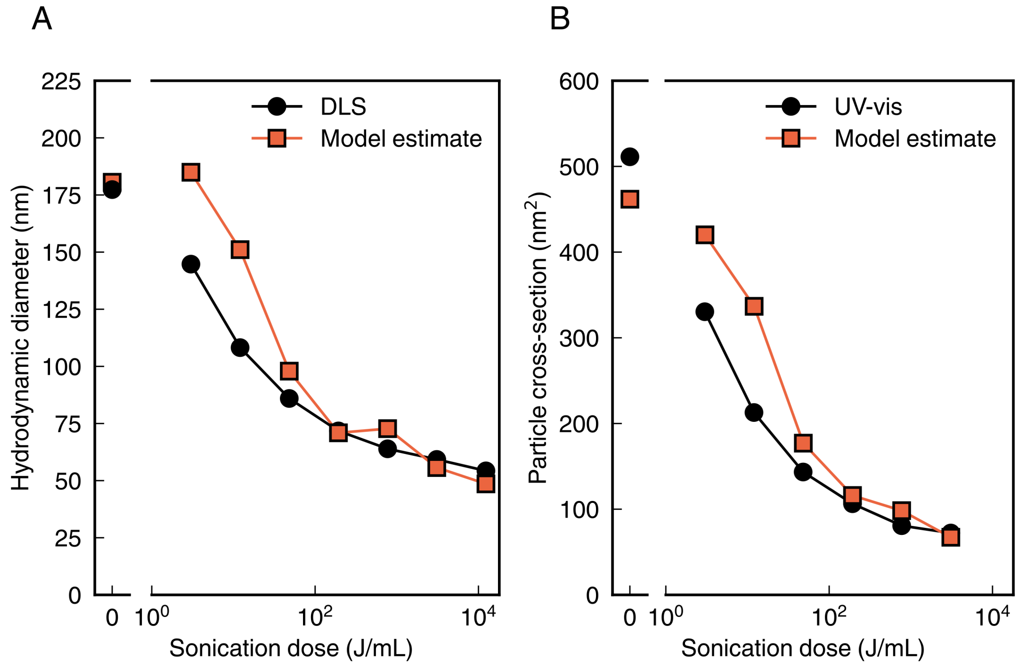

Supplementary Figure 15: Comparison of experimentally-measured ensemble size properties and estimated values based on individual particle sizes in TEM images. (**A**) Hydrodynamic diameter (**B**) Particle cross-section.

10 Correlating CNC phase behaviour with particle morphology

For cylindrical particles forming a lyotropic liquid crystal phase, the concentrations of the isotropic-biphasic and biphasic-anisotropic phase boundaries (ϕ_0 and ϕ_1 respectively), and the position of the midpoint of the biphasic region ($\phi_m = (\phi_0 + \phi_1)/2$), are expected to be inversely proportional to the cylinder aspect ratio, as shown by Onsager and subsequent authors (13; 14).

As shown in Supplementary Figure 16A, the biphasic midpoint ϕ_m is fairly well-described by a linear relation with inverse 3D aspect ratio (i.e. $\phi_m \propto \alpha_{3D}^{-1}$), in line with theoretical predictions. However, only ϕ_1 is observed to increase with sonication dose, while ϕ_0 appears to remain constant. In terms of re-scaled volume fraction $\phi\alpha_{3D}$, only $\phi_1\alpha_{3D}$ significantly increases with sonication dose (Supplementary Figure 16B). This broadening can be attributed to an increase in the coefficient of variation (relative polydispersity) in 3D aspect ratio $\tilde{\sigma}_\alpha = \sqrt{(\langle\alpha^2\rangle - \langle\alpha\rangle^2)/\langle\alpha\rangle^2}$ (note the 3D subscript has been omitted for clarity). Supplementary Figure 16C shows the variation in $\tilde{\sigma}_\alpha$ with re-scaled volume fraction, which shows trends similar to a previous theoretical study on length-polydisperse colloidal rods (15).

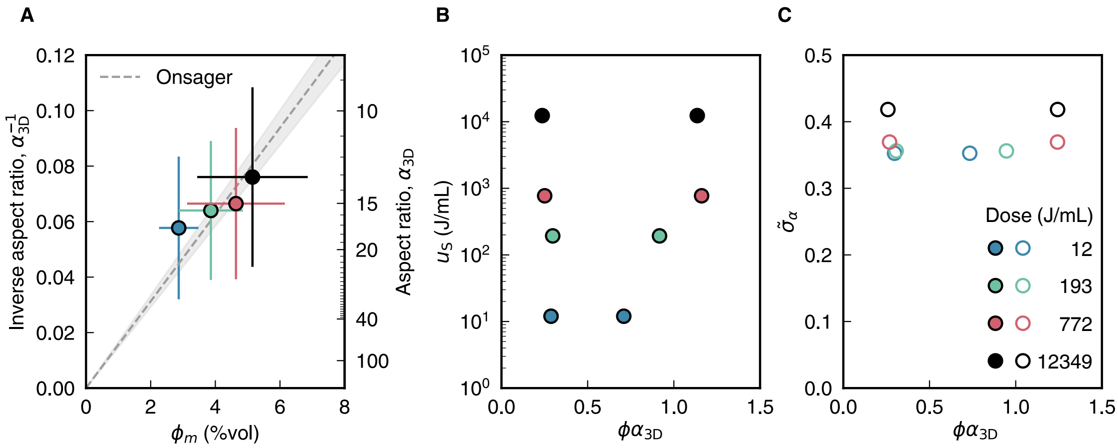

Supplementary Figure 16: (**A**) Correlation between inverse 3D aspect ratio and midpoint of the biphasic region. Error bars in biphasic midpoint indicate inter-quartile range. Grey line indicates linear fitting $\phi_m \propto \alpha_{3D}^{-1}$ forced to pass through the origin. (**B**) Biphasic boundary points (ϕ_0, ϕ_1) plotted versus re-scaled volume fraction $\phi\alpha_{3D}$. (**C**) Aspect ratio polydispersity $\tilde{\sigma}_\alpha$ plotted versus re-scaled volume fraction $\phi\alpha_{3D}$. Colour scheme in the legend of (C) is used for all subfigures.

11 Classification of CNC particles

The classification of CNC particles into four classes (A, B, C, D) is based on their shape properties observed in TEM images, as described in the article. The relative number fraction of each particle class ($X = A, B, C, D$) in the overall population is given by

$$\mathcal{N}_X = \frac{N_X}{\sum_X N_X} \tag{18}$$

where N_X is the number of particles in class X. The relative number fraction for each sonication dose in shown in Supplementary Figure 17

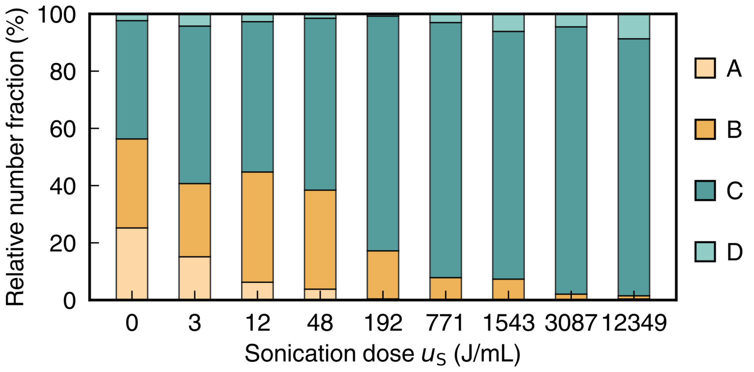

Supplementary Figure 17: Relative number fraction $\mathcal{N}_X$ for each particle class $X = A, B, C, D$.

Alternatively, the relative volume fraction of each particle class can also be calculated

$$\mathcal{V}_X = \frac{V_X}{\sum_X V_X} \tag{19}$$

where V_X is the total volume of all particles in class X. To calculate V_X, the volume of each CNC in class X (index j) is estimated using the expression

$$v_j = A_j \langle T \rangle_j \tag{20}$$

where A_j is the projected area observed in the TEM image and $\langle T \rangle_j$ is the estimated mean particle thickness (see section 8 for details). The total volume is then given by

$$V_X = \sum_{j \in X} v_j \tag{21}$$

Note that the relative volume fractions are normalised such that $\sum_X \mathcal{V}_X = 1$. The absolute volume fraction of particles of class X (in the suspension) is given by $\phi_X = \mathcal{V}_X \phi_{\mathrm{CNC}}$. The relative volume fractions versus sonication dose is shown in the article.

The differences in morphological properties between the sub-populations can be clearly seen in histograms for each population. Histograms for particle box length L_b and 3D aspect ratio α_{3D} of each class are shown in Supplementary Figure 18. Histograms for the cross-section aspect ratio expressed as $\alpha_{\mathrm{XS,AE}}$ and $\alpha_{\mathrm{XS,b}}$ (defined in Supplementary Table 6) are shown in Supplementary Figure 19. Histograms for the 2D isoperimetric quotient and estimated 3D isoperimetric quotient are shown in Supplementary Figure 20.

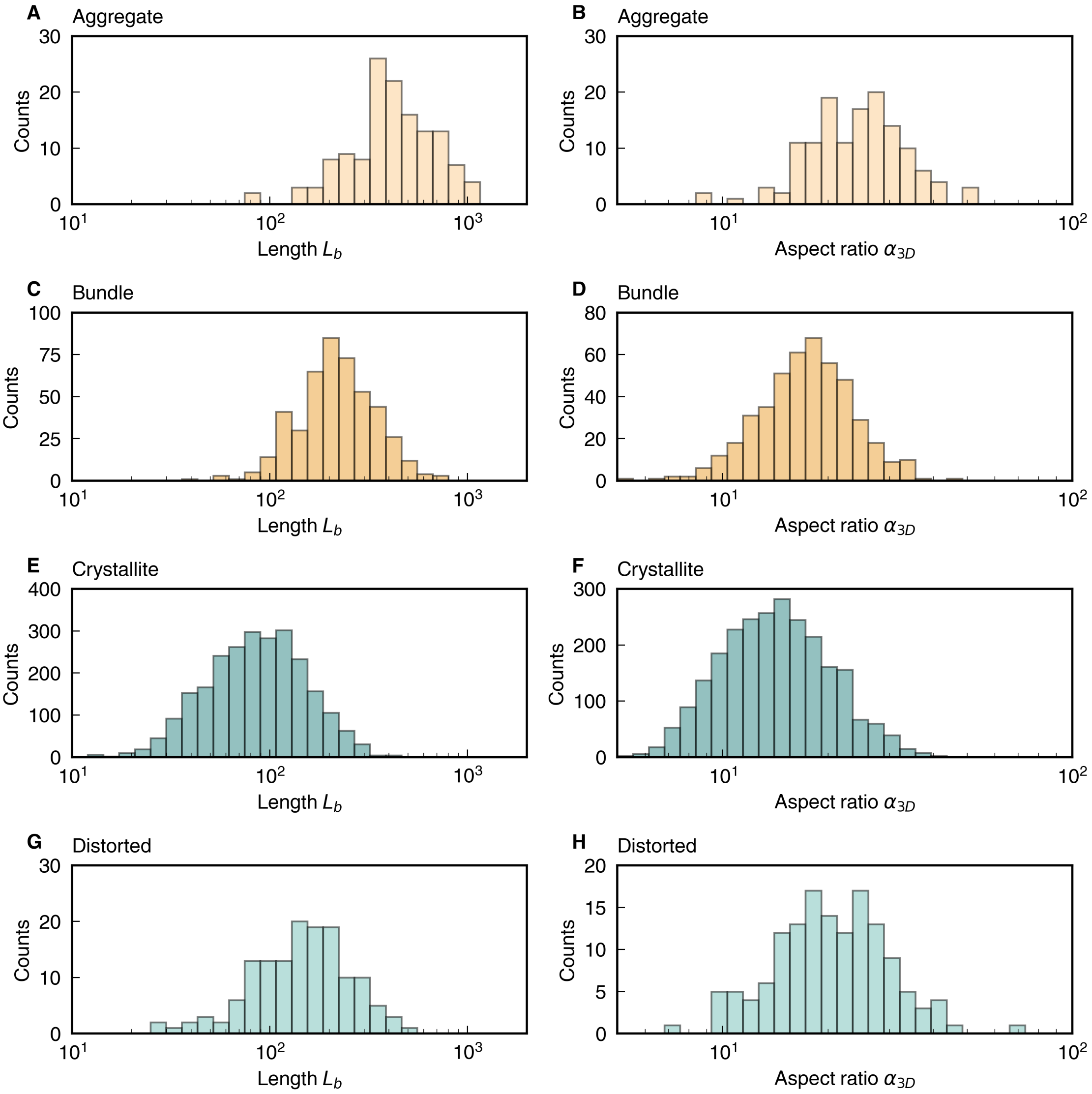

Supplementary Figure 18: Histograms for particle length L_b and 3D aspect ratio α_{3D} for each particle class.

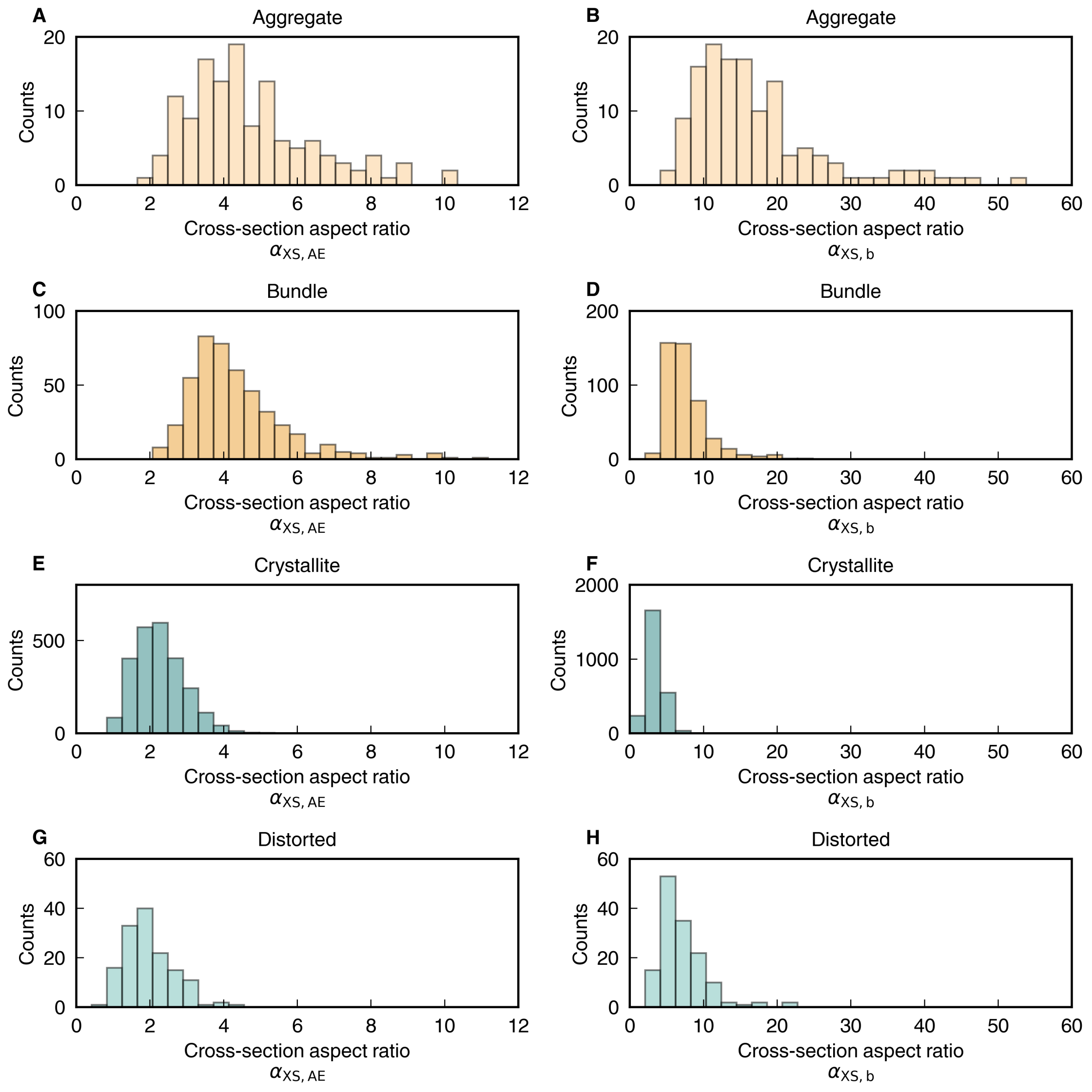

Supplementary Figure 19: Histograms for $\alpha_{XS,AE}$ and $\alpha_{XS,b}$ for each particle class.

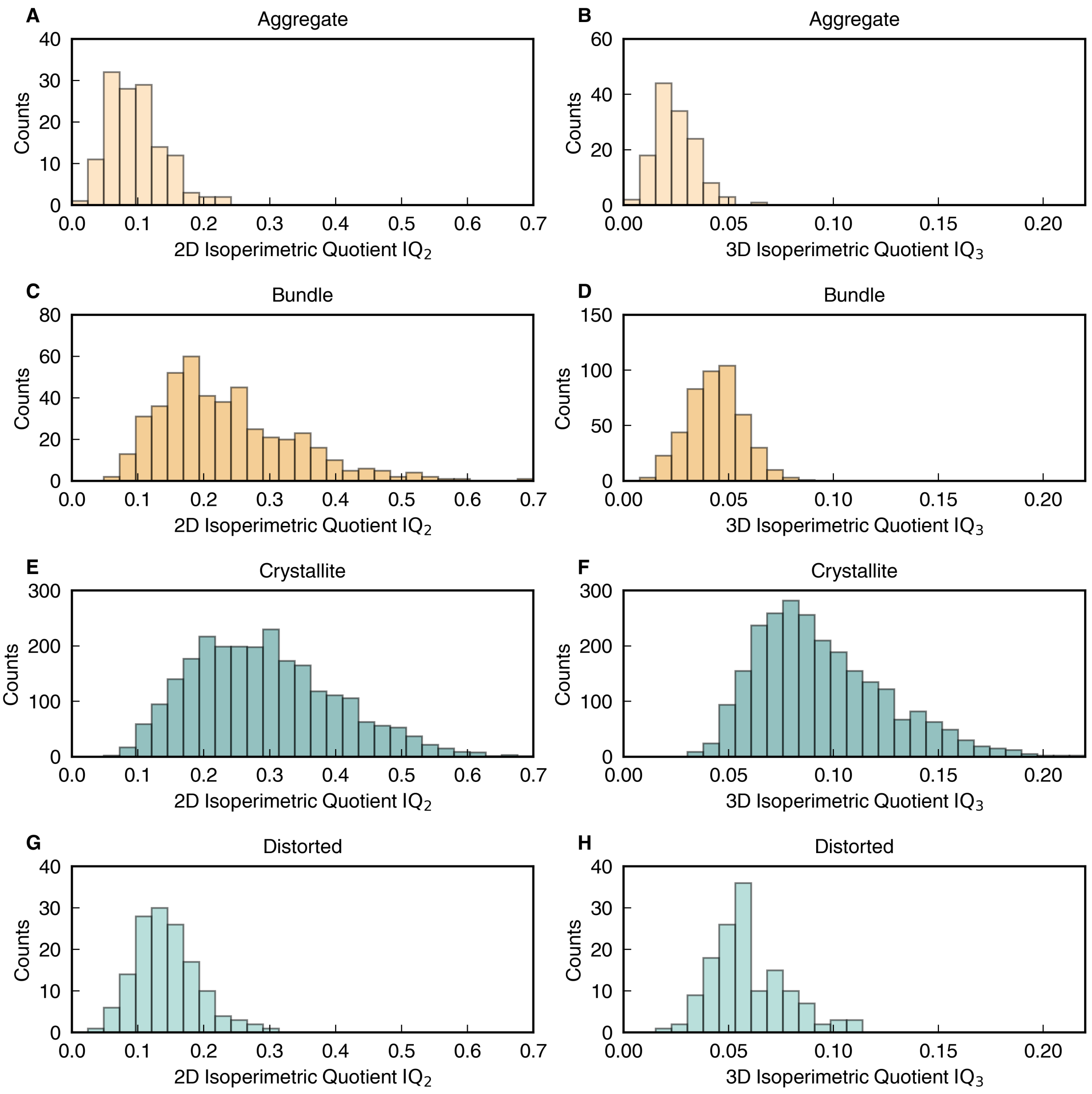

Supplementary Figure 20: Histograms for IQ_2 and IQ_3 for each particle class.

12 Supplementary Methods

12.1 Dynamic light scattering (DLS)

DLS samples were prepared with CNC (0.1 wt%) suspended in 1 mM NaCl solution, and passed through a 0.8 µm cellulose acetate syringe filter before pouring into disposable plastic cuvettes. DLS measurements were performed with a commercial setup (Malvern Zetasizer Nano ZS) using 633 nm illumination and collecting back-scattered (173°) light. Samples were measured at 25 °C after a delay of 300 s to allow for thermal equilibration before data collection, which was performed in three runs of at least ten measurements per run.

12.2 Electrolytic conductivity and pH measurement

The conductivity and pH of CNC suspensions were determined using a platinum 2-pole conductivity probe (InLab 752-6MM, Mettler Toledo) or pH probe (InLab Micro Pro-ISM, Mettler Toledo) respectively. All measurements were performed at room temperature.

12.3 Optical spectroscopy of CNC photonic films

Left-circular polarised (LCP) reflectance spectra of CNC photonic films were obtained using the microscope setup used to collect polarised optical microscopy images (see Methods and Figure 1 of the main article). The light reflected from the sample passed through an LCP analyser, composed of an achromatic quarter-wave plate and linear polariser (Thorlabs WP25M-UB), before being transmitted to a UV-vis spectrometer (AvaSpec-HS2048, Avantes) *via* an optical fibre (600 μm core diameter, FC-UV600-2-SR, Avantes). Reflection spectra were normalised to a silver mirror (Thorlabs, PF10-03-P01).

12.4 Scanning electron microscopy (SEM) of CNC photonic films

Fully dry CNC films were pulled apart to expose their cross-sections. The films were then mounted onto steel stubs using conductive carbon tape. Samples were coated with platinum to a nominal thickness of 10 nm using a sputter coater (Quorum Q150T ES) to ensure good conduction of electrons. Micrographs were taken using a TESCAN MIRA3 FEG-SEM system using an acceleration voltage of 5 kV and a working distance of 3-6 mm.

12.5 Conductometric titration of CNC suspensions

In a typical titration procedure, 2.0 g of 2.0 wt% CNC suspension was added to 200 mL of 0.5 mM NaCl solution. The acidic CNC suspensions (with H^+ counter-ions on the CNC sulfate half-ester groups) underwent extensive dialysis against deionised water prior to measurement. An automatic titrator (Metrohm 856) was used to inject NaOH solution (10 mM) in 5 μL increments, while continuously recording the suspension conductivity. The surface charge per CNC dry mass (mmol/kg) was determined from the first equivalence point of the titration curve, obtained by a manual piecewise linear fitting.

12.6 Zeta potential of CNC suspensions

The CNC samples used for zeta potential measurements were identical to those used for DLS measurements (Supplementary Methods, section 12.1). The zeta potential was estimated from the measured electrophoretic mobility using the Smoluchowski limit of the Henry equation ($\kappa R \gg 1$,
$F(\kappa R) = 1.5$), which is the conventional choice for CNC suspensions (16). Measurements were performed in three batches of at least 50 runs each.

12.7 UV-vis transmission spectroscopy of CNC suspensions

UV-vis transmission spectra were obtained using a commercial spectrophotometer (Cary 4000). CNC samples were prepared at 0.1 wt% and measured in a quartz cuvette (Hellma 100-10-40)

with 10 mm path length.

12.8 Cryogenic transmission electron microscopy (cryoTEM) of CNC suspensions

Cryogenic transmission electron microscopy (cryoTEM) imaging was carried out using a JEM 3200FSC field emission microscope (JEOL) operated at 300 kV in bright field mode with an Omega-type zero-loss energy filter. The images were acquired with a Ultrascan 4000 CCD camera (Gatan) and processed with Gatan Digital Micrograph software (version 1.83.842).

Vitrified samples were prepared using EM GP2 Automatic Plunge Freezer by placing a 4-5 µL droplet of sample solution onto plasma-cleaned 300-mesh lacey carbon copper grids in a 90% humidity atmosphere, then blotted with filter paper for 0.5 - 1.5 seconds, followed by immediate immersion into an ethane/propane mixture at −170 °C. The samples were then cryo-transferred to the microscope, where their temperature was maintained at −187 °C.

12.9 Atomic force microscopy (AFM) of CNC suspensions

Atomic force microscopy (AFM) images of the cellulose nanocrystals were acquired at ambient conditions using a scanning probe microscope (Agilent 5500 SPM) in tapping mode with an AFM probe (OTESPA-R3). A square of mica (2 cm^2) was freshly cleaved to obtain a mirror smooth surface. A droplet (100 µL, 0.1 wt%) of poly-L-lysine (P8920, Sigma, M_W = 150-300 kDa) was deposited for 1 minute, after which it was rinsed off with deionised water and dried under nitrogen gas flow. Then, the CNC sample (150 µL, 0.001 wt%, pH = 3) was deposited and incubated for 3 minutes after which it was washed off with deionised water and dried under nitrogen gas flow. Finally, the sample was dried in the oven for 30 minutes at 50 °C and then kept at ambient conditions in a closed dish before the measurements. Scans were typically performed over a 4x4 μm^2 area with 2048 points per line at 0.6 Hz to acquire the final images with ~2 nm resolution.

CNC particle height statistics were extracted from AFM images using Gwyddion software (17). The images were processed in six steps: (1) removing the background height variation using a fifth-order polynomial fitting (2) aligning rows by median values (3) healing scars (4) flattening the base (5) setting the zero offset to the median image height and finally (6) Gaussian filtering with 2 pixel resolution.

To estimate the AFM tip diameter, AFM images were also obtained for spherical gold nanoparticles with diameter $D \approx 15$ nm. The tip diameter was estimated by the difference between the max height of the particle and the apparent lateral diameter (Supplementary Figure 11C). Measurement on 38 particles gave $D_{tip} = 14.2 \pm 1.1$ nm (Supplementary Figure 21)

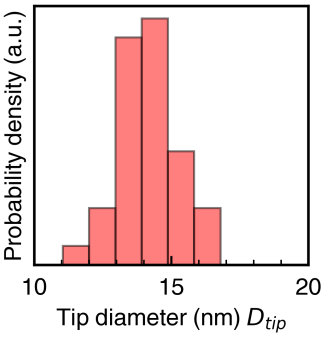

Histogram of estimated tip diameter $D_{\mathrm{tip}} = D_{\mathrm{eff}} - D$ for spherical gold nanoparticles.

12.10 Transmission electron microscopy (TEM) shape analysis

As previous authors have noted, there is great variability in CNC size data obtained from TEM images, even for two users manually measuring identical experimental data (18). The tracing method used in this work is therefore described in detail below to aid comparison with other works.

CNC particles as observed in TEM images were manually traced to extract size and shape properties. Tracing was performed on a touch-screen device using a stylus and the "Freehand Selections" tool in Fiji/imageJ. In negatively-stained TEM images, CNCs generally appear as bright spindle-shaped objects surrounded by a dark "halo" or outline created by the accumulation of staining agent around the particle. The CNC particle was taken to be the entire bright area within this dark outline. The particle areas were filled white on the original image and then selected by thresholding the image.

We found that suspending the CNCs in an aqueous pH 3 solution of sulphuric acid dramatically reduced the occurrence of artefacts due to particle aggregation on the dried TEM grid, in agreement with previous work (19). Therefore, any single continuous bright region, regardless of shape, was assumed to be a single CNC particle. In particular, even if the shape could be interpreted as two elongated particles overlapping, the shape was still assumed to be a single particle.

The traced shapes were analysed using the ImageJ Shape Filter plugin, filtering for all particles larger than 70 nm^2 to eliminate any artefacts due to pixel noise. The data were exported in .csv format and further processed using a custom Python script.

References

[1] A. G. Dumanli, *et al.*, *ACS Applied Materials & Interfaces* **6**, 12302 (2014).

[2] P. Vanysek, *CRC hand book of chemistry and physics* pp. 5–92 (1993). Publisher: CRC press.

[3] S. Beck, J. Bouchard, R. Berry, *Biomacromolecules* **12**, 167 (2011).

[4] X. M. Dong, T. Kimura, J.-F. Revol, D. G. Gray, *Langmuir* **12**, 2076 (1996).

[5] R. M. Parker, *et al.*, *Advanced Materials* **30**, 1704477 (2017).

[6] G. Guidetti, H. Sun, A. Ivanova, B. Marelli, B. Frka-Petesic, *Advanced Sustainable Systems* **5**, 2000272 (2021). _eprint: https://onlinelibrary.wiley.com/doi/pdf/10.1002/adsu.202000272.

[7] T. Abitbol, D. Kam, Y. Levi-Kalisman, D. G. Gray, O. Shoseyov, *Langmuir* **34**, 3925 (2018).

[8] M. Shimizu, *et al.*, *Macromolecular Rapid Communications* **37**, 1581 (2016).

[9] E. F. Casassa, *The Journal of Chemical Physics* **23**, 596 (1955). ISBN: 0021-9606 Publisher: American Institute of Physics.

[10] M. E. Carr Jr, J. Hermans, *Macromolecules* **11**, 46 (1978). ISBN: 0024-9297 Publisher: ACS Publications.

[11] M. M. Tirado, C. L. Martínez, J. G. de la Torre, *The Journal of Chemical Physics* **81**, 2047 (1984). Publisher: American Institute of Physics.

[12] G. Schumacher, T. G. M. v. d. Ven, *Journal of the Chemical Society, Faraday Transactions* **87**, 971 (1991). Publisher: Royal Society of Chemistry.

[13] L. Onsager, *Annals of the New York Academy of Sciences* **51**, 627 (1949).

[14] A. Stroobants, H. N. W. Lekkerkerker, T. Odijk, *Macromolecules* **19**, 2232 (1986).

[15] H. H. Wensink, G. J. Vroege, *The Journal of Chemical Physics* **119**, 6868 (2003).

[16] E. J. Foster, *et al.*, *Chemical Society Reviews* **47**, 2609 (2018). Publisher: The Royal Society of Chemistry.

[17] D. Nečas, P. Klapetek, *Open Physics* **10**, 181 (2012). Publisher: De Gruyter Open Access.

[18] A. Brinkmann, *et al.*, *Langmuir* **32**, 6105 (2016).

[19] M. Kaushik, C. Fraschini, G. Chauve, J.-L. Putaux, A. Moores, *Transm. Electron Microsc. Theory Appl* (2015).
